# Rainfall extremes drive cocoa yield losses across the tropics

**DOI:** 10.1073/pnas.2606123123

**Published:** 2026-09-14

**Authors:** Anna Lea Albright, Rebecca Berkoh-Oforiwaa, Peter Huybers

**Affiliations:** ^a^ Department of Earth and Planetary Sciences, Harvard University, Cambridge, MA 02138; ^b^ Department of Physics, University of Ghana, Legon, Accra – LG 25, Ghana

**Keywords:** crops, climate, human-centered climate information, rainfall extremes, agriculture

## Abstract

There is a growing need for human-centered weather and climate forecasts that provide actionable information to farmers and other stakeholders. Cocoa, which sustains the livelihoods of nearly six million smallholder farmers, illustrates this need: Recent production shortfalls have threatened incomes, yet their underlying causes remain unclear, with explanations invoking both climatic and nonclimatic factors. Here we show that heavy rainfall, rather than temperature or mean water availability, is the dominant climatic constraint on cocoa yields across major producing regions. Using newly available district-level production records from Ghana combined with daily rainfall and temperature data, we find that excess wet-season rainfall and dry-season drought together explain 68% of interannual yield variability. National yield data from Ecuador and Indonesia also give a consistent negative response to heavy rainfall, indicating a common mechanism across the tropics, likely linked to damage and disease risks associated with intense precipitation during key phenological stages. Because rainfall extremes covary with El Niño and other sea surface temperature patterns, and there is a robust physical expectation of increases in rainfall extremes with warming, these results provide a physical basis for risk forecasts. By shifting attention from average climate conditions to extreme precipitation, we highlight an underappreciated constraint on a perennial tropical crop and identify opportunities for adaptation and forecast-informed decision-making.

## Significance

Nearly six million smallholder farmers depend on cocoa for their primary income. Production shortfalls threaten this livelihood, yet the causes of these shortfalls are poorly understood. This study shows heavy rainfall is the dominant climatic constraint on cocoa yields across producing regions. By identifying heavy rainfall during flowering and early pod development as a recurrent source of yield loss, this work shifts attention from average climate trends to extreme events relevant for management and adaptation. Because rainfall extremes are partly predictable from large-scale patterns such as El Niño, and are expected to increase with warming, these results highlight an opportunity to assess crop-climate risks. Extreme precipitation may represent an underappreciated risk for perennial tree crops under increasingly variable hydroclimate conditions.

**Fig. 1.**
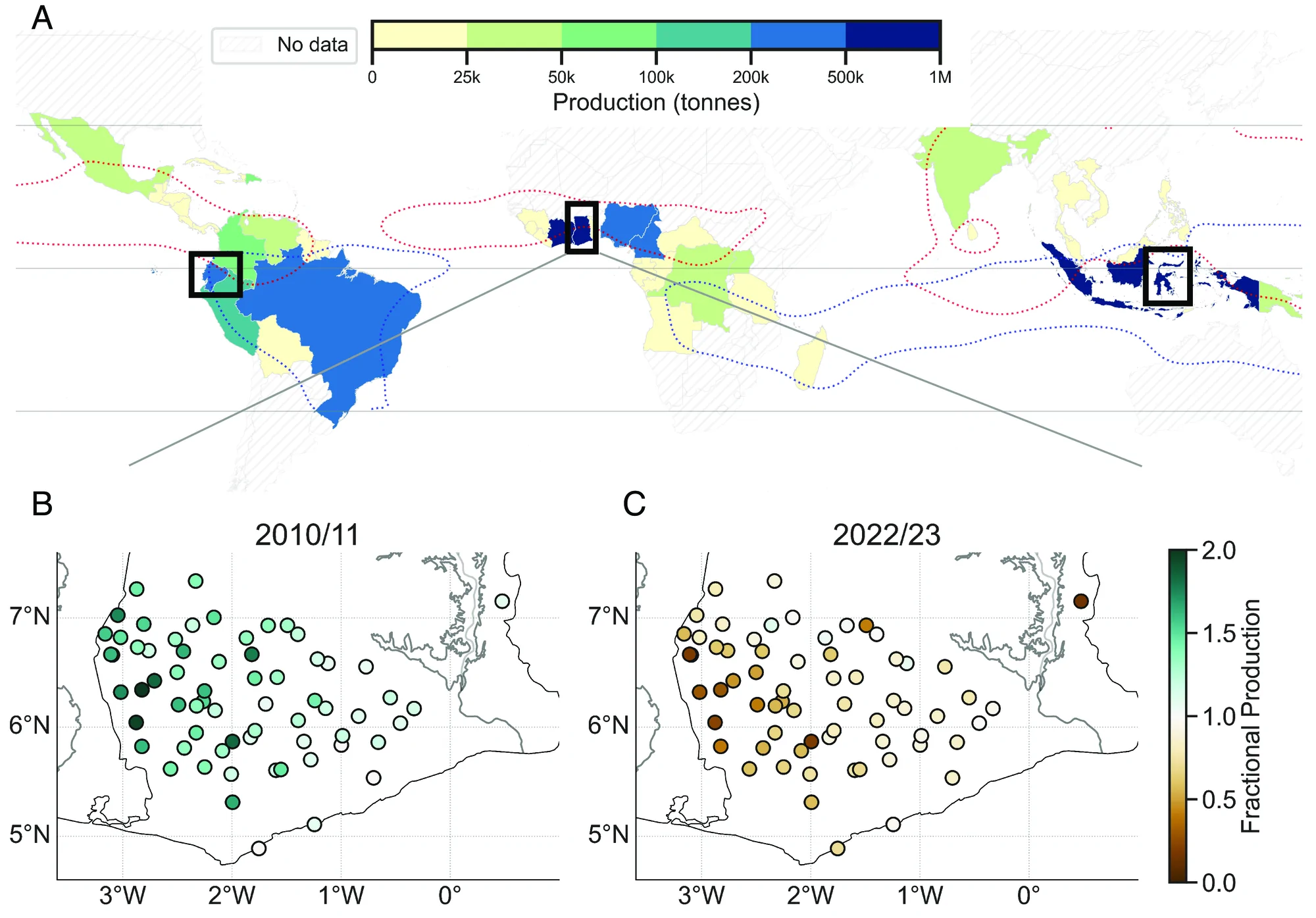
(*A*) Cocoa production data (2023) from the Food and Agriculture Organization of the United Nations (FAO) (5), overlain with climatological contours of the Intertropical Convergence Zone (ITCZ) position in February (blue) and August (red) estimated as 30-y averages from the PERSIANN rain dataset. (*B* and *C*) Coherent spatial variations in fractional production anomalies across 66 districts in Ghana for the 2010/2011 and 2022/2023 seasons.

Cocoa is an understory tree native to the equatorial forests of South America where it was first domesticated about 5000 years ago (1, 2). Today, four tropical countries with the consistently warm and moist conditions that allow cocoa to thrive (3, 4) account for the vast majority of global cocoa production (5) (Fig. 1*A*): Côte d’Ivoire (∼40%), Ghana (∼20%), Indonesia (∼12%), and Ecuador (∼7%). In the 2023/2024 season, West Africa experienced harvest losses of up to 40% (5), following several years of reduced production. These shortfalls threaten the primary source of income for approximately two million smallholder farmers in West Africa and six million farmers worldwide, while also affecting an estimated 40 to 50 million people whose livelihoods are tied to cocoa-reliant industries (6, 7).

Proposed causes for the recent production shortfalls in West Africa include aging trees (8), gold mining (9), smuggling (10), reduced fertilizer access (4), and low pollination (11). The extent to which nonclimatic versus climatic factors drive cocoa yields, however, remains uncertain. Agronomic studies generally identify insufficient rainfall and water stress as a dominant limitation on yield (1, 12–14). Although cocoa can tolerate one to three dry months per year (15), its shallow root system makes it highly sensitive to moisture deficits (16, 17). Other studies suggest that high temperatures may become, or are already, as limiting as water availability in shaping future cocoa suitability (18, 19), with yields declining when maximum temperatures exceed 32 to 33 °C (19). Weather and climate factors have therefore been widely invoked, but mostly in relation to heat, drought, or mean rainfall rather than to extreme rainfall.

A growing literature shows that climate variability explains a substantial fraction of yield anomalies for major annual crops, with temperature extremes generally exerting stronger and more consistent impacts than precipitation variables (20, 21). When precipitation effects are detected, they are often nonlinear and timing-dependent: Moderate rainfall extremes can benefit yields, whereas the heaviest events reduce production through waterlogging, crop damage, and disease pressure (22–24). Across multiple crops and regions, drought typically emerges as the dominant rainfall-related constraint (25, 26).

By contrast, the role of heavy rainfall remains poorly resolved for perennial tropical tree crops, despite their long lifespans, distinct phenology, and sensitivity to disease dynamics. Whether insights derived from annual crops extend to systems such as cocoa, coffee, and fruit trees—which provide the main source of income to millions of smallholder farmers throughout the tropics—remains an open question. Cocoa provides a useful test case for addressing this gap: It is a perennial tropical tree crop whose yields depend on distinct flowering and pod-development stages that coincide with seasonal environmental variability (1, 13).

Here we investigate the origins of interannual variability in cocoa yields and whether these origins are consistent across the tropical cocoa belt. Using two decades of newly available district-level data from the Ghana Cocoa Board paired with daily rainfall and temperature observations, we identify the weather signals driving production fluctuations. We find that rainfall extremes explain the majority of interannual production variability. Short-duration rainfall extremes during vulnerable phenological stages are the dominant driver of yield losses in this tropical perennial tree crop. This finding contrasts with crop–climate studies that emphasize temperature extremes or seasonal-mean water availability. Applying the same framework to Indonesia and Ecuador indicates a consistent association between rainfall extremes and yield variability. Understanding the causes of historical production shortfalls informs prediction of future agricultural risks and dovetails with the goal of developing “human-centered weather forecasts” (27)—systems designed to provide actionable climate information for farmers and other stakeholders.

## Developing a Cocoa Yield Model for Ghana

We focus first on Ghana, which offers newly available district-level production records from the Ghana Cocoa Board, covering 66 administrative districts from the 2000/2001 to 2022/2023 seasons (*Materials and Methods*). We estimate the 2023/2024 season anomaly based on national production data from the USDA Ghana Cocoa Report (9) as a 20% reduction relative to the prior season.

To isolate year-to-year productivity deviations from the long-term expansion in cultivated area and changes in farming practices, we fit a linear trend to each district’s annual production and divide the time series by this trend; examples are given in *SI Appendix*, Figs. S1 *B* and *C*, and S2. We define these detrended data as “fractional production anomalies,” and they serve as a proxy for yield variations (*Materials and Methods*). Fractional production anomalies have similar statistical properties to the detrended logarithm of yield, which is commonly used in agricultural studies, under conditions where year-to-year variations in planted area are small relative to variations in production (*Materials and Methods*). Supporting the use of fractional production as a yield proxy, cocoa trees typically require three to five years to bear fruit and can remain productive for 30 to 40 y, or even up to 60 y under optimal conditions (28), indicating slower changes in planted area.

Spatial patterns of fractional production anomalies provide important insight into the primary drivers of interannual cocoa production. If factors like gold-mining encroachment, soil heterogeneity, or differences in access to agricultural inputs were dominant, anomalies would be expected to exhibit spatially localized patterns. Instead, as illustrated for two contrasting seasons (Fig. 1 *B* and *C*), fractional production anomalies across cocoa-producing districts in Ghana are largely coherent in sign. That is, in some years, such as 2010/2011, fractional production anomalies are generally greater than one (above the linear trend) across districts, while in other years, such as 2022/2023, anomalies are generally less than one, below the trend.

Year-to-year, production anomalies vary coherently across districts, with strong agreement between individual districts and the district-median production (*SI Appendix*, Fig. S1 *D* and *E*). The Pearson correlation between each district and the district median averages 0.66 (interquartile range of 0.60 to 0.77), with 67% of districts exceeding the mean. In this negatively skewed distribution, only a small number of districts deviate from this pattern, most notably the easternmost district (*SI Appendix*, Fig. S1 *D* and *E*). The high degree of spatial coherence is consistent with an important role for large-scale external drivers, such as regional weather variability, in shaping interannual cocoa production fluctuations.

### A National-Level Yield Model.

To identify the dominant meteorological drivers of interannual cocoa yield variability in Ghana, we begin by establishing the relevant phenological context. For Ghana, we define the production year, t, as the cocoa season beginning in March of calendar year t and ending in February of year t+1 (*Materials and Methods*). Cocoa flowering and the initiation of new pods (cherelles) peak during the major rainy season, when rainfall exerts strong control on floral bud and cherelle production (29). This period also coincides with the main period of pollinator activity (29). Pods initiated during the first rainy season contribute to the main cocoa harvest, which typically spans October–March and accounts for 70 to 80% of annual production, with recent seasons frequently extending through May (9). Accordingly, climate windows are mapped to the production year in which the associated pods are harvested (*Materials and Methods*).

We apply a suite of complementary statistical learning approaches to identify dominant predictors, including least absolute shrinkage and selection operator (LASSO) regression, elastic net regression, and a random forest model (*Materials and Methods*). Candidate climate predictors (*SI Appendix*, Table S3) are derived from daily rainfall (30) and daily temperature (31). They include mean and 95th-percentile daily rainfall from the TAMSAT Pan-African dataset (32) and indicators of cool and heat stress defined by daily minimum temperatures below 18^°^C and daily maximum temperatures above 32 ^°^C, following physiological thresholds for cocoa (3). To retain seasonal structure while limiting predictor dimensionality, climate variables are aggregated into two-month windows aligned to the cocoa crop year (March–February, with January–February assigned to the preceding year). Results are qualitatively similar when individual months are used instead.

Across all three modeling approaches, a consistent subset of predictors emerges as dominant. Both lasso and elastic net identify the 95th percentile of daily rainfall during the April–June primary wet season and mean rainfall during the November–February dry season as the most influential predictors of interannual production variability (*SI Appendix*, Table S4). These variables are also ranked highly by the random forest model, despite its ability to represent nonlinear interactions, indicating that the identified relationships are robust across linear and nonlinear formulations. Consistent with the cocoa phenological cycle, these two predictors are interpreted as acting on distinct developmental stages: The April–June wet-season rainfall metric affects flowering and early pod development, whereas the November–February dry-season metric reflects moisture limitation during later pod filling and maturation. Because these stages feed into the main harvest from approximately October until March or May, both climate windows are assigned to the same production year t (*Materials and Methods*).

**Fig. 2.**
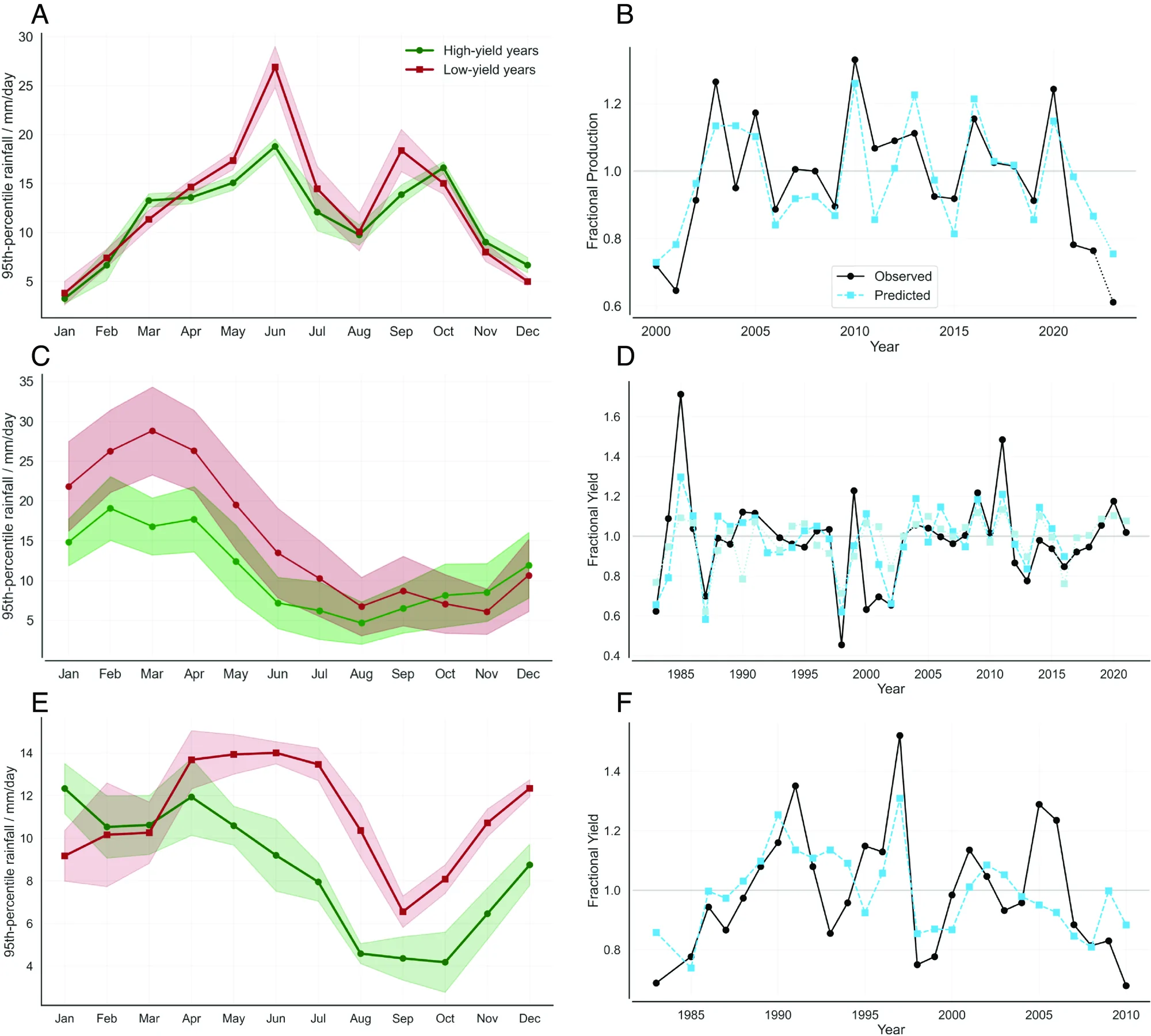
Cocoa production and yields in relationship to heavy wet-season rainfall and mean dry-season rainfall for Ghana (*Top*, *A* and *B*); and to heavy wet-season rainfall only in Ecuador (*Middle*, *C* and *D*) and Indonesia (Sulawesi) (*Bottom*, *E* and *F*). *Left* column (*A*, *C*, and *E*): monthly 95th percentile of daily rainfall, averaged across the five seasons with the highest (green) and five seasons with the lowest (red) fractional production or yield; shading indicates the SE. For Ghana and Indonesia, the wettest three months are April–June, and for Ecuador, the wettest months are February–April. *Right* column (*B*, *D*, and *F*): observed (black) and predicted (blue, dotted) time series; for Ghana (*B*), the last year is the 2023/2024 season, dotted because it is estimated from national rather than district-level data. For Ecuador (*D*), the light blue predictor is PERSIANN satellite data, whereas the medium blue predictor is REGEN rain gauge data ending in 2016.

As an illustration, compositing monthly 95th-percentile rainfall for the five years with the highest and the five years with the lowest fractional production anomalies shows that lower yielding years are associated with heavier rainfall during the first rainy season and reduced rainfall during the subsequent dry season (Fig. 2*A*).

Based on this consistent predictor selection, we formulate a parsimonious linear model:

[1] $$\begin{array}{c} P\left(t\right)=\alpha_{0}+\alpha_{w}R_{w}\left(t\right)+\alpha_{d}R_{d}\left(t\right)+\epsilon \left(t\right), \end{array}$$

where P(t) denotes the median fractional production anomaly in year t, $R_{w}\left(t\right)$ is the monthly 95th percentile of daily rainfall, averaged across the three months of the primary wet season, April–June, and $R_{d}\left(t\right)$ is mean daily rainfall during the November–February dry season. Using the district-median fractional production anomaly and spatial-mean rainfall (*Materials and Methods*), this model explains 68% of interannual variance over the 2000/2001–2023/2024 seasons (Fig. 2*B* and Table 1). These seasonal rainfall variations exhibit strong spatial coherence, with cross-correlations between the country-wide median and individual districts averaging 0.82±0.12 for wet-season extreme rainfall and 0.93±0.08 for mean dry-season rainfall. Although wet-season extremes and dry-season rainfall deficits have often co-occurred in recent years, there is no discernible correlation over the full period of record (r=−0.14 in TAMSAT from 2000/2001 to 2023/2024 and r=0.34 in PERSIANN; *Materials and Methods*).

**Table 1.** Rainfall regression results

Model	$\alpha_{w}$ (*P*)	$\alpha_{d}$ (*P*)	$R^{2}$	AIC	BIC
Ghana				
Base model (TAMSAT)	−0.093 (0.001)	0.11 (<0.001)	0.68	−34	−31
Dry-season soil moisture	−0.10 (<0.001)	0.11 (<0.001)	0.68	−34	−31
Dry-season dry days	−0.10 (0.001)	−0.087 (0.003)	0.57	−27	−24
Consecutive dry days	−0.10 (0.001)	−0.078 (0.011)	0.52	−25	−21
Wet-season soil moisture	−0.0077 (0.815)	0.11 (0.002)	0.38	−18	−15
*Wet-season median rain (TAMSAT)*	−0.034 (0.258)	0.12 (0.001)	0.46	−22	−18
Extreme rain (PERSIANN)	−0.014 (0.70)	0.107 (0.006)	0.32	−16	−13
Extreme rain (CHIRPS)	−0.027 (0.41)	0.12 (0.001)	0.40	−19	−16
Indonesia (Sulawesi)				
Base model	−0.13 (<0.001)	—	0.42	−20	−17
*Wet-season median rain*	−0.083 (0.034)	—	0.17	−10	−7.6
Ecuador				
Extreme rain only (REGEN)	−0.17 (<0.001)	—	0.52	−23	−20
*Wet-season median rain (REGEN)*	−0.12 (0.002)	—	0.25	−8.2	−5.2
Extreme rain (PERSIANN)	−0.12 (0.001)	—	0.29	−16	−12
Extreme rain (CHIRPS)	−0.11 (0.001)	—	0.27	−16	−13
Extreme + dry-season mean rain (REGEN)	−0.18 (<0.001)	0.061 (0.029)	0.61	−28	−23
Extreme + dry-season dry days (REGEN)	−0.17 (<0.001)	−0.058 (0.036)	0.60	−28	−23
Extreme + consecutive dry days (REGEN)	−0.18 (<0.001)	−0.019 (0.52)	0.55	−23	−19

Coefficients for wet season ($\alpha_{w}$) and dry-season ($\alpha_{d}$) predictors are shown with *P*-values in parentheses. Model fit statistics [$R^{2}$, Akaike Information Criterion (AIC), and Bayesian Information Criterion (BIC)] are reported for Ghana, Sulawesi (Indonesia), and Ecuador. All predictors are standardized. Italicized rows denote models using the 50th percentile rather than the 95th percentile of rainfall. Data periods are 2000/2001–2023/2024 for Ghana, 1983–2010 for Sulawesi, and for Ecuador 1983–2016 using REGEN and 1983–2021 using PERSIANN and CHIRPS. For Ghana and Ecuador, additional models include dry-season rainfall metrics as a second predictor. For Ghana, soil moisture is derived from TAMSAT using the JULES land model (33). This analysis tests the recurrence of climatic predictors across regions rather than the transferability of fitted coefficients.

Model robustness is further assessed using leave-one-year-out cross-validation (LOOCV). While LOOCV does not preserve temporal ordering (unlike the time-series cross-validation used in model selection), it provides a useful test of sensitivity to individual years. The resulting out-of-sample $R^{2}$ of 0.57 (compared to 0.68 in-sample), together with stable regression coefficients across iterations and consistent results from an expanding cross-validation, indicates that the identified rainfall controls are not driven by overfitting or isolated extreme years.

### Validation Through Alternative Model Formulations.

To assess the robustness of Eq. **1** and validate its underlying assumptions, we examine alternative formulations across three dimensions: 1) functional form and loss functions, 2) alternative climate metrics, and 3) inclusion of temperature predictors.

#### Alternative functional forms.

The parsimonious linear model captures the direction of most interannual fluctuations, but underestimates the amplitude of both the most extreme positive and negative anomalies. Residuals, computed as observed minus predicted fractional production, increase in magnitude with observed production (r=0.57, P=0.004; *SI Appendix*, Fig. S4), indicating a state-dependent bias. The attenuation of extremes is symmetric and expected in regression models that minimize squared error, which shrinks extreme observations toward the mean. If we instead use third-order polynomials to relate wet- and dry-season rainfall to yield, we obtain a higher adjusted $R^{2}=0.66$ and smaller residuals (*SI Appendix*, Fig. S5).

A variety of other loss functions and nonlinear formulations are, however, less effective. Median regression (using an L1 loss function) produces a similar overall residual structure, but increases the magnitude of residuals and strengthens their trend with respect to observed values; it also lowers the explained variance (adjusted $R^{2}=0.60$, *SI Appendix*, Fig. S5), as expected. Random forest models and lasso regression techniques produce similar residual structure to the linear model (*SI Appendix*, Fig. S5). These results indicate that while higher-order models can modestly improve fit, changes in model formulation do not qualitatively alter the identified relationships or substantially change predictive skill when accounting for model complexity.

#### Alternative climate metrics.

To test whether the choice of specific rainfall metrics influences our conclusions, we examine alternative formulations. For wet-season extremes, besides different high quantiles (*Materials and Methods*), we consider metrics describing rainfall onset, frequency, and temporal clustering. The 95th percentile of daily rainfall is significantly anticorrelated with rainy-season onset after April 1 (r=−0.54, P<0.01) and strongly correlated with the number of heavy-rainfall days exceeding 20 mm d^−1^ during April–June (r=0.82, P<0.01). A metric describing clustering of heavy rainfall events—the maximum number of consecutive days exceeding 15 mm d^−1^—is correlated with the 95th percentile (r=0.54, P<0.01) but is driven by a small number of years and does not outperform the percentile-based metric. That is, the 95th percentile is correlated with the rainy season beginning earlier and having more heavy-rainfall days and longer runs of consecutive heavy-rainfall days, and none of these other indicators of heavy rainfall make for better predictors.

For dry-season moisture availability, motivated by recent evidence that dry days and consecutive dry periods play a key role in cocoa-relevant drought impacts in West Africa (14), we examine dry-day counts, maximum dry-spell duration, and mean dry-season soil moisture derived from the TAMSAT land-surface modeling framework (33) (*Materials and Methods*). The soil moisture variable represents plant-available root-zone moisture estimated by a land-surface model driven primarily by rainfall and tuned with satellite observations. All alternative metrics are strongly correlated with mean dry-season rainfall and do not substantially improve explanatory power (Table 1), coefficient stability, or spatial patterns. Of the alternatives, replacing mean dry-season rainfall with mean soil moisture yields a comparable fit (same $R^{2}=0.68$, AIC, and BIC; Table 1). However, soil moisture remains highly correlated with rainfall (r=0.83), reflecting their shared precipitation forcing. For parsimony and consistency across regions where soil moisture data are unavailable, we retain mean dry-season rainfall as the primary drought indicator.

#### Temperature effects.

Temperature-based stress metrics, including cumulative exposure to daily maximum temperatures above 32 ^°^C and daily minimum temperatures below 18 ^°^C, were ranked lower by both the regularized linear regressions and random forest models (*SI Appendix*, Table S4). Adding the two highest-ranked temperature predictors, heat exposure in September/October and March/April, to Eq. **1** does not improve model performance. Adjusted $R^{2}$ remains unchanged, while AIC and BIC increase relative to the rainfall-only specification (*SI Appendix*, Table S6), indicating no gain in explanatory power after penalizing for additional complexity. Across model formulations and evaluation criteria, rainfall extremes consistently outperform temperature-based predictors.

These validation exercises confirm that the simple two-predictor model in Eq. **1** captures the dominant climatic controls on interannual cocoa production variability in Ghana. While alternative formulations can provide modest improvements in specific metrics such as the adjusted $R^{2}$ or address particular residual structures, they consistently support the central finding: In Ghana, excessive wet-season rainfall and dry-season moisture limitation are the primary climatic constraints on cocoa yields.

### District-Level Models Provide Further Insight Into Mechanisms.

**Fig. 3.**
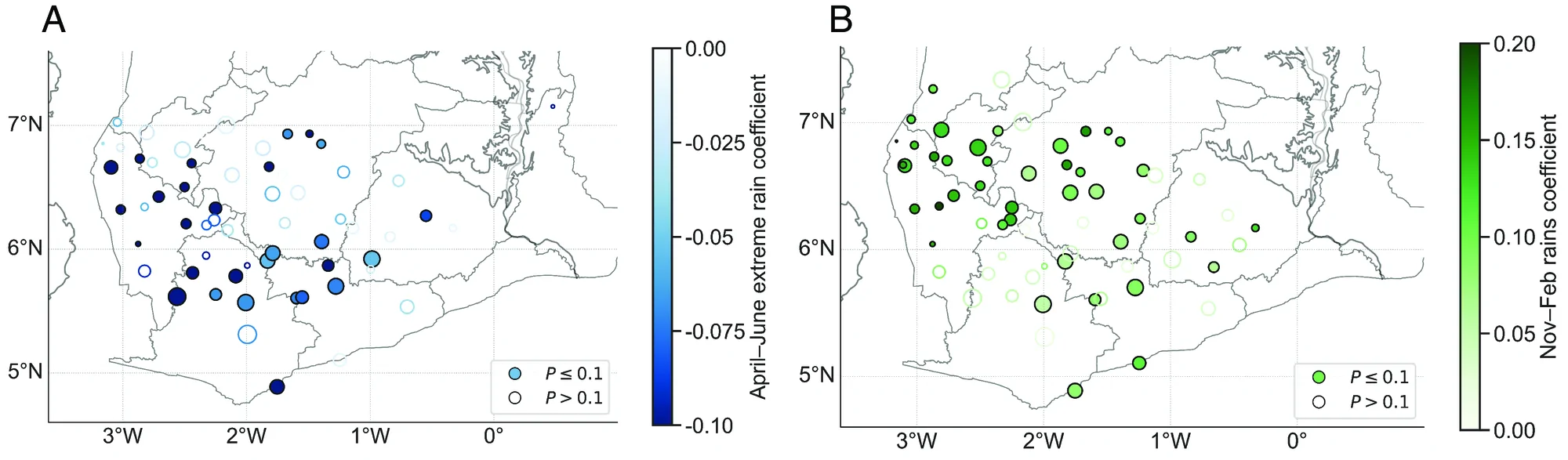
Analysis of district-level cocoa production anomalies. Linear regressions are repeated using nearest-neighbor rainfall values for each district with 1° × 1° TAMSAT rainfall data; panels show the estimated coefficients for heavy rainfall (*A*) and for mean dry-season rainfall (*B*) across 66 Ghanaian districts. Filled markers indicate statistical significance at the 10% level (P≤0.1); open markers indicate P>0.1. The marker size is proportional to 2022/2023 production in tonnes.

Moving from national to district-scale analysis reveals spatial differentiation in climate sensitivities that provides additional mechanistic insight. District-level regressions indicate that in northern transitional zones between forest and savanna, dry-season rainfall is the stronger predictor of yield variability (Fig. 3*B*), whereas in southern districts heavy rainfall during the main wet season has a greater effect (Fig. 3*A*). If we define northern districts as above $6^{{}^{\circ}}$N, 76% of northern districts show moderately significant (P≤0.1) associations with dry-season rainfall, whereas below $6^{{}^{\circ}}$N only 33% of districts show such significance. Conversely, 62% of districts in the south are significant with respect to extreme rainfall, but only 33% in the north. Repeating the analysis with 0.25° rather than 1° rainfall data gives similar results (*SI Appendix*, Fig. S7).

For both 1° and 0.25° data, about a quarter of the districts exhibit low skill for both dry-season rainfall and wet-season heavy rainfall. Identifying the drivers of production anomalies in these districts remains an important open question, possibly emphasizing nonclimatic factors such as small-scale gold mining. The effects of smuggling are possibly more prominent in 2010/2011, with elevated production in western Ghana (Fig. 1*B*) suggestive of smuggling into the country, when cocoa trade out of Côte d’Ivoire was disrupted by conflict. In contrast, the 2022/2023 season (Fig. 1*C*) saw smuggling out of Ghana, both to Côte d’Ivoire and Togo, possibly driven by elevated international cocoa prices and relatively lower farmgate producer prices in Ghana (34). Price-driven smuggling incentives are likely lower in more liberalized cocoa economies like Ecuador and Indonesia, where farmgate prices track global markets more closely, and we consider these countries next.

Results in Ghana are sensitive to the choice of rainfall product. The dry-season coefficient is robust across products, but CHIRPS and PERSIANN give insignificant heavy-rainfall coefficients at both the national (Table 1) and district (*SI Appendix*, Fig. S8) scales. The upper tails of the CHIRPS and PERSIANN daily rainfall distributions are compressed relative to Ghanaian rain gauge observations (*SI Appendix*, Fig. S9 and *Materials and Methods*). Ref. 35 reaches a similar conclusion in an independent validation against 38 rain gauge stations in Ghana and Zambia: TAMSAT achieves the highest probability of detection for moderate daily rainfall, between 50% and 80% across nearly all Ghanaian stations, while all three products have low skill for heavy and violent rain.

## Rainfall Extremes Consistently Drive Yield Variability Across Tropical Regions

To assess whether similar climatic relationships extend beyond Ghana, we apply the same modeling framework and candidate predictor set to Ecuador and Indonesia (Sulawesi). For both countries, we use national-level cocoa yield data from the FAO (5), detrended to reduce the influence of nonclimatic factors such as cultivar improvement and pest management and to isolate interannual variability driven by climate (*Materials and Methods*). Ecuador and Sulawesi each experience a single dominant rainy season (Fig. 2 *C* and *E*). As in Ghana, wet-season extreme rainfall is defined using the 95th percentile of daily precipitation, and multiple dry-season moisture metrics are evaluated (*Materials and Methods*).

In Ecuador, penalized linear regressions and a random forest applied to the same candidate predictor set used for Ghana (*SI Appendix*, Table S3) consistently identify wet-season extreme rainfall as the dominant driver of interannual cocoa yield variability (*SI Appendix*, Table S5). This result is robust across rainfall products: Analyses using REGEN, PERSIANN, and CHIRPS all yield negative and statistically significant coefficients for wet-season extreme rainfall (Table 1). A parsimonious linear model based solely on the 95th percentile of rainfall during the three wettest months (February–April) explains 29% and 27% of yield variance over 1983–2021 when driven by PERSIANN and CHIRPS, respectively. Using the REGEN rain gauge dataset, which requires restricting the analysis to 1983–2016, increases the explained variance to 52% (Fig. 2 *C* and *D*). Applying the same temporal restriction to PERSIANN and CHIRPS yields explained variances of 28% and 34%, respectively, compared with REGEN’s 52%, indicating that differences among rainfall products, rather than record length, account for most of the variation in model skill. Despite well-documented discrepancies among rainfall datasets (36, 37), the negative association between heavy wet-season rainfall and cocoa yields emerges as a consistent feature.

Including a dry-season moisture availability metric, identified as a secondary predictor by the regularized regressions and random forest, yields a modest additional improvement in model fit. Using the REGEN dataset, adding either mean June–September rainfall or dry-day counts increases the explained variance from 52% to approximately 60% and reduces AIC and BIC, whereas a metric based on consecutive dry days does not consistently improve performance (Table 1). Smaller and less consistent improvements are observed when using CHIRPS and PERSIANN. This mixed behavior contrasts with the consistently negative yield response to excessive wet-season rainfall, which emerges robustly across all rainfall products.

In Sulawesi, rainfall explains less yield variance, reflecting both data limitations and substantial nonclimatic changes in production. Penalized regressions do not consistently select individual predictors, in line with a low signal-to-noise ratio in the yield record. Nevertheless, simple correlations indicate that wet-season extreme rainfall exhibits the strongest association with yields, and a random forest ranks the 95th percentile of April–June rainfall as the most important predictor of interannual variability. A single-predictor linear model using April–June extreme rainfall explains 20% of yield variance over 1983–2020, increasing to 42% when the analysis is restricted to 1983–2010 (Fig. 2*F* and Table 1). This improvement likely reflects reduced nonstationarity prior to widespread crop replacement, as farmers increasingly shifted from cocoa to oil palm and cloves in response to disease pressure and market incentives beginning in the 2000s (38). If crop replacement dynamics are not fully captured in national harvested-area estimates, they can obscure climate–yield relationships in later years.

Applying the same candidate predictors and model-selection workflow independently to Ecuador and Indonesia again identifies wet-season rainfall extremes as the strongest predictor of interannual cocoa yield variability. This recurrence across regions indicates a consistent climatic sensitivity rather than a location-specific artifact. Furthermore, as in Ghana, fitting more flexible nonlinear models in Ecuador and Indonesia gives results consistent with the simple linear formulation.

The results from Ecuador and Indonesia show that when the same modeling framework and candidate predictors are applied across independent cocoa-producing regions, wet-season rainfall extremes consistently exhibit a significant negative association with interannual cocoa yield variability, despite substantial differences in data quality, production systems, and nonclimatic influences relative to West Africa. This convergence across regions indicates that extreme rainfall represents a robust and recurring climatic constraint on cocoa yields.

## Heavy Rainfall Can Reduce Yields Through Multiple Biotic and Abiotic Pathways

Heavy rainfall emerges as the strongest predictor of cocoa yield losses. During the wet season, extreme rainfall provides substantially greater explanatory power than mean or median rainfall, as well as soil moisture metrics (Table 1), which could be interpreted as closer proxies for persistent humidity or waterlogged conditions. Although elevated relative humidity and leaf wetness are known to promote fungal infection (39–41), and field studies link both humidity and rainfall to reduced yields (42–44), our results suggest that the occurrence of heavy rainfall events, captured in daily weather data, best signals risk to yields.

We outline three mechanisms that link heavy rainfall to reduced cocoa yields. First, cocoa flowers during the rainy season, overlapping with peak pollinator activity (29). Heavy rainfall during the main wet season therefore directly affects the flowers and young pods that determine the subsequent harvest: Exposed reproductive structures are vulnerable to mechanical damage, and intense rain and wind can induce flower and cherelle abortion (45, 46). Second, excessive rainfall could interfere with cocoa pollination (11, 47).

Third, extreme rainfall promotes fungal disease. Field observations indicate a lag of approximately one week between rainfall events and the onset of black pod rot (43). In West Africa, primary infections of black pod disease (*Phytophthora*) typically begin near the end of the first rainy peak and can persist for months (48, 49). Heavy rainfall facilitates both the reactivation of soilborne inoculum (44, 50) and the dispersal of spores via rain splash (51). During a severe outbreak in Ghana’s Akomadan district, initial infections appeared predominantly on pods near the ground (50), supporting soil reservoirs and splash-mediated spread, as well as possible invertebrate or tool-mediated transmission (44, 50). Supporting these mechanisms, cocoa black pod disease has been shown to increase with the frequency of rainfall events rather than with total rainfall amounts (42), aligning with our finding that short-duration rainfall extremes are more predictive of yield losses than seasonal-mean wetness.

We note that cocoa swollen shoot virus, transmitted by mealybug insects, also severely curtails yields in West Africa (52) and is speculated to covary with drought conditions (53). This association requires further study but is not the focus here.

The extreme rainfall finding has two immediate implications. First, adaptation to rainfall extremes can target i) interruption of splash-driven transmission, ii) protection of flowers and cherelles, iii) optimized planting density, and iv) well-timed fungicide application; the latter two strategies were shown to narrow cocoa yield gaps (54). In practice, “utilizing weather data for pest and disease surveillance allows for targeted application of fertilizers and insecticides, maximizing their effectiveness” (46), underscoring the value of such human-centered weather and crop studies for developing targeted adaptation strategies. Second, improving forecasts of rainfall extremes, from weather to seasonal to longer timescales, would provide actionable climate information; we discuss this potential for rainfall forecasting next.

## Outlook: Predictability and Trends in Rainfall Extremes

The foregoing analysis raises a follow-on question: Are recent rainfall extremes and production shortfalls manifestations of internal climate variability, or do they signal an emerging climate shift driven by greenhouse gas warming?

**Fig. 4.**
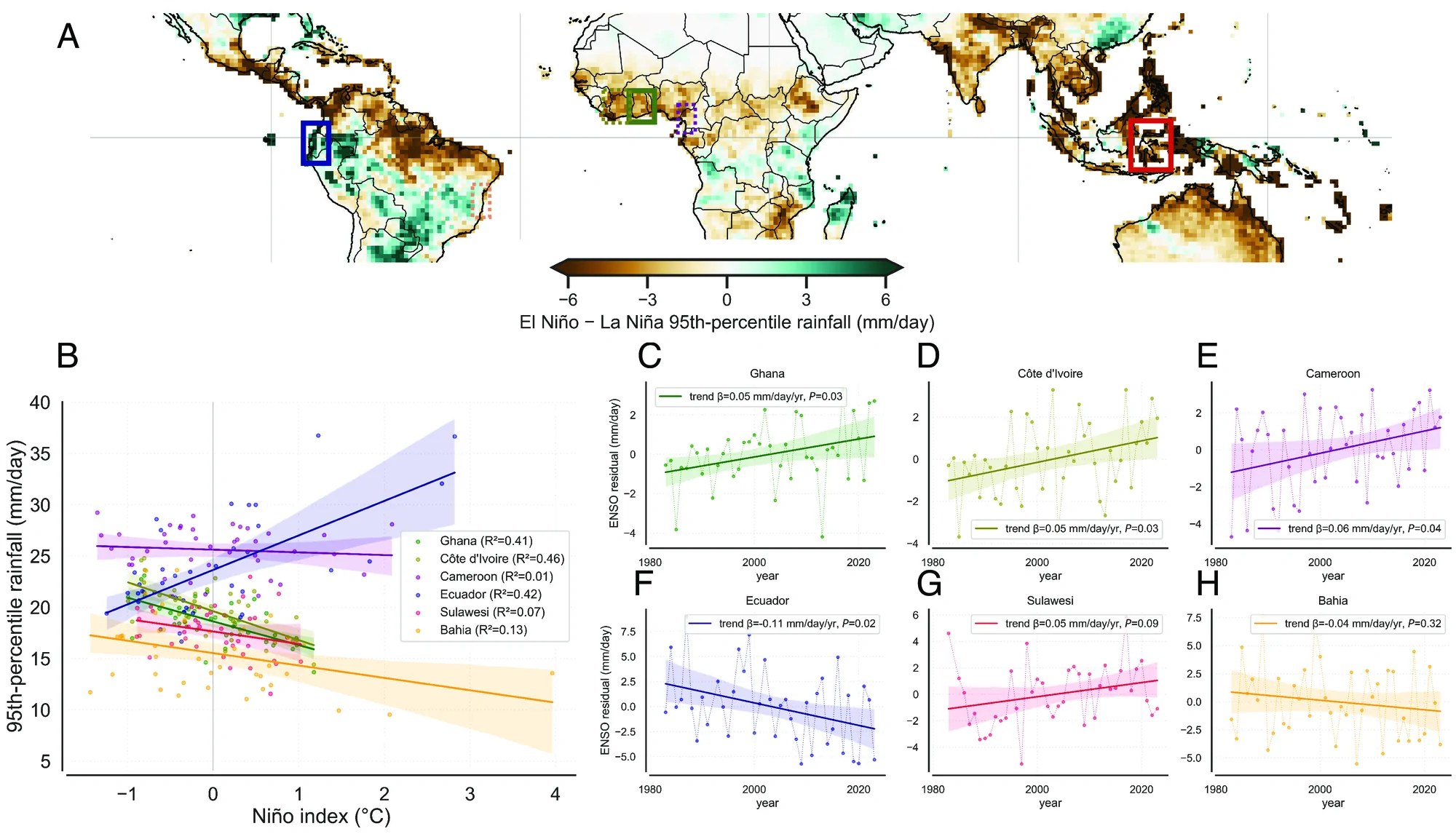
ENSO variability and decadal trends in heavy rainfall. (*A*) February–January 95th percentile rainfall anomalies averaged across El Niño (detrended Niño 3.4 index ≥ 0.5 ^°^C) minus La Niña (detrended Niño 3.4 index ≤ −0.5 ^°^C) years; rainfall in panel *A* is from the REGEN dataset over 1983–2016. (*B*) 95th percentile rainfall during the peak rainy season against the Niño index (Niño 3.4 for Ghana, Côte d’Ivoire, Cameroon, and Sulawesi; Niño 1 + 2 for Ecuador and Bahia). In Ghana, Côte d’Ivoire, and Cameroon, the Niño index is calculated using months that precede the rainfall period by one month; for the other regions, the Niño index and rainfall periods cover the same months. For panels *B–H*, 95th percentile rainfall is computed from TAMSAT over Ghana, Côte d’Ivoire, and Cameroon, and from PERSIANN over Ecuador, Sulawesi, and Bahia, in each case spanning 1983–2023. (*C–H*) Linear trends in 95th percentile rainfall, with regression coefficients and P-values indicated, after removing ENSO effects using linear regression. For Sulawesi, Indonesia (red), April–June is shown; ENSO explains little of the April–June rainfall variance there ($R^{2}\approx 0.07$), although it strongly modulates October–December rainfall ($R^{2}\approx 0.62$).

As a null hypothesis, we first consider internal climate variability. On interannual timescales, across cocoa-producing regions, wet-season heavy rainfall is strongly modulated by the El Niño–Southern Oscillation (ENSO), with 41% and 42% of variance explained in Ghana and Ecuador (Fig. 4*B* and *SI Appendix*, Table S7). During El Niño events, West Africa and Indonesia typically experience reduced rainfall, while coastal Ecuador and East Africa receive more (Fig. 4*A*). ENSO’s predictability on one- to two-year timescales (55) provides a physical basis for yield-risk forecasts. In addition to ENSO, independent tropical Atlantic sea surface temperature variability (the Atlantic Niño) explains 21 to 34% of variance in extreme rainfall for cocoa-growing regions in Ghana, Côte d’Ivoire, and Cameroon (*SI Appendix*, Table S7). ENSO more weakly modulates Ghana’s November–February dry-season mean rainfall (Niño 3.4, $R^{2}=0.11$ over 1983–2023 and $R^{2}=0.18$ over 2000/01–2023/24). Together, these modes account for much of the interannual rainfall variability associated with cocoa yield variability and define the null expectation of internally driven fluctuations.

After removing the ENSO-driven internal variability signal, residual wet-season extremes still exhibit decadal trends from 1983 to present day. Historical wet-season rainfall in West Africa exhibits an increasing trend (Fig. 4*C*–*E*), consistent with physical expectations (56) and with climate model projections of intensifying heavy rainfall under global warming (57, 58). Gauge records from 97 stations in southern West Africa (1970–2014) show rising wet-season rainfall intensity (59). Satellite observations confirm intensifying mesoscale convective systems across the region since 1983, associated with strengthening surface temperature gradients and wind shear (60), providing a physical mechanism for the increased heavy rainfall that threatens cocoa production. This upward trend is concerning for cocoa production in West Africa, as it suggests that heavy rainfall may continue to increase. Not all cocoa-growing regions show this expected signal over the historical period, however: in Ecuador, the ENSO residual of wet-season heavy rainfall has decreased since 1983 (Fig. 4*F*). The residual trend in Sulawesi is weakly positive (Fig. 4*G*), and it is insignificant in Bahia, Brazil (Fig. 4*H*). These regional contrasts could reflect shifts in the mean position of the Intertropical Convergence Zone, migrating north (e.g., away from Ecuador) toward a faster-warming Northern Hemisphere (61), but this hypothesis requires further study.

Our findings indicate that the 2023/2024 and earlier West African cocoa production shortfalls are not isolated events and suggest an emerging risk associated with intensifying rainfall extremes under global warming. Although cocoa is often considered mainly vulnerable to drought and heat stress, our results indicate that excessive rainfall during key phenological stages can be just as important and, in some regions, represents the dominant constraint on yields. Given robust physical expectations that heavy rainfall events will intensify as the climate warms (e.g., ref. 56), this mechanism may become an increasingly important limitation on cocoa production with continued warming.

More broadly, these results highlight the need to reconsider how climate risks to agriculture are assessed. Many crop–climate analyses emphasize temperatures or seasonal-mean water availability, yet our findings demonstrate that short-duration rainfall extremes, when aligned with vulnerable phenological stages, can exert disproportionate impacts on yields. By identifying heavy rainfall during flowering and early pod development stages as a recurrent source of yield loss, this work shifts attention from average climate trends to extreme events that are directly relevant for disease management, planting practices, and seasonal decision-making.

Because rainfall extremes are, moreover, in part, predictable from large-scale climate modes such as El Niño and the Atlantic Niño, these results highlight an opportunity to develop actionable, human-centered climate forecasts for perennial crops. A similar mechanism may be relevant for other tropical tree crops and rainfed systems that depend on steady rainfall during flowering and early fruit development, including coffee, oil palm, and fruit trees. Identifying crop-specific sensitivities to rainfall extremes will be essential for improving seasonal risk forecasts, guiding adaptation strategies, and supporting livelihoods in a future characterized by increasingly intense hydrological variability.

## Materials and Methods

### Data Sources.

#### Daily rainfall data: Sources and spatial averages.

We employ daily rain data from both ground-based rain gauges and satellites at a consistent $1^{{}^{\circ}}\times 1^{{}^{\circ}}$ spatial resolution, accessed using the Frequent Rainfall Observations on GridS (FROGS) database (30), as summarized in *SI Appendix*, Table S2. Daily-mean, rather than monthly-mean, data are needed to calculate the heavy rainfall metric as described in the main text. For TAMSAT, we also consider rainfall at $0.25^{{}^{\circ}}\times 0.25^{{}^{\circ}}$, accessed via the TAMSAT data subset service.

We define regional averages based on latitude and longitude bounds corresponding to the primary cocoa-growing areas, consistent with area information in ref. 62. For Ghana, we use 5° to 7°N and 3.5° to 0.5°W; for Ecuador, 3.5°S to 1.5°N and 80.5° to 78.5°W; and for Sulawesi (Indonesia), 6.5°S to 1.5°N and 119.5° to 125.5°E. We select Sulawesi as representative of Indonesia given that approximately 80% of cocoa is produced on this island (63). These regional bounds are relatively coarse and, in the case of Sulawesi, encompass both lowland alluvial plains and higher-elevation areas and therefore do not represent regional heterogeneity. Due to the lack of subnational yield data, we focus on these larger spatial averages of rainfall as they are better suited for capturing large-scale climate signals such as ENSO and reduce noise. While spatial aggregation improves comparability with district- and national-scale production data, it necessarily smooths highly localized rainfall extremes. Such field-scale events, which may contribute to the most severe local yield failures, are therefore not fully resolved and may contribute to residual variance and muted responses in extreme years.

For Ghana, in addition to TAMSAT, we also consider CHIRPS and PERSIANN daily rainfall. We do not consider REGEN for Ghana since this dataset ends in 2016 (*SI Appendix*, Table S2), and the Ghana cocoa record begins in 2000. PERSIANN appears less reliable for West Africa in that it shows trends toward diminished heavy rainfall during the first rainy season over recent decades, a finding that diverges from increases in heavy rainfall found in southern Ghana rain gauge observations from 1970 to 2014 (59). We further discuss differences between rainfall products and their implications for the dry-season mean rainfall and wet-season heavy rainfall coefficients in *Heavy rainfall metric*.

#### Soil moisture data.

For Ghana, root-zone soil moisture is from the TAMSAT soil moisture (TAMSAT-SM) dataset, a new long-term, operational product developed for agricultural drought monitoring across Africa (33). TAMSAT-SM provides estimates of plant-available root-zone soil moisture from 1983 to the present at 0.25° spatial resolution. The dataset is generated using the JULES land-surface model, forced primarily by TAMSAT rainfall estimates and additional meteorological variables from the NCEP reanalysis, and calibrated using SMAP satellite soil moisture observations. As such, TAMSAT-SM represents modeled root-zone moisture availability (or moisture stress) relevant to crop water demand, rather than a direct in-situ observation. Mean dry-season soil moisture is computed by averaging monthly TAMSAT-SM values over the dry-season months November–February, and we also consider mean soil moisture averaged over the April–June primary rainy season.

### Climate Metrics.

#### Heavy rainfall metric.

We quantify heavy rainfall as the monthly 95th percentile of spatially averaged daily precipitation across the three wettest months of each cocoa-growing season (*SI Appendix*, Table S1), where the 95th percentile is computed for each month and then averaged.

To assess robustness, we also consider alternative definitions of extreme rainfall, including the 90th and 99th percentiles which are highly correlated with the 95th percentile ($R^{2}=0.83$ and $R^{2}=0.62$, respectively), and the mean or sum of days exceeding a fixed climatological threshold (e.g., the 95th percentile for each month averaged over a 2000–2020 baseline). We therefore focus, for simplicity, on the 95th percentile averaged for the three wettest months, while noting that alternative approaches to quantifying extreme rainfall yield qualitatively similar results. In the main text (*Alternative climate metrics*), we describe metrics capturing rainfall onset, frequency, and temporal clustering. Rainfall onset is defined as the first day between 1 April and 30 June on which a centered 5-d rainfall total reached at least 25 mm and was followed by a second consecutive 5-d total exceeding 25 mm, ensuring persistence of wet conditions. Onset timing was expressed as days since 1 April; years without a qualifying event were excluded. Heavy-rainfall days are defined as the sum of days exceeding 20 mm d^−1^ during April–June. To quantify clustering of heavy rainfall events, we also consider the maximum number of consecutive days exceeding 15 mm d^−1^ in April–June, with similar results if considering 20 mm d^−1^.

In terms of spatially averaging and then calculating the 95th percentile, or vice versa, both the magnitude and variability are similar for Ghana and Ecuador. For Indonesia, the variability is similar, but first spatially averaging reduces the magnitude compared to finding the 95th percentile in each grid box for each month and then averaging across months and across space. Compared to Ghana and particularly Ecuador, where topographic zones are more clearly separable, averaging across Sulawesi’s complex topography with alluvial plains and highlands in the gridded data is likely to cause a lower magnitude seasonal-mean rainfall value.

In Ghana, in addition to TAMSAT, we also consider CHIRPS and PERSIANN daily rainfall. All three products yield a significant dry-season rainfall coefficient of consistent sign and magnitude, but the wet-season heavy-rainfall coefficient is strongly significant only in TAMSAT ($\alpha_{w}=-0.093$, P=0.001); it is attenuated and insignificant in CHIRPS ($\alpha_{w}=-0.027$, P=0.41) and PERSIANN ($\alpha_{w}=-0.014$, P=0.70; Table 1). The disagreement is therefore specific to heavy rainfall (*SI Appendix*, Fig. S8). PERSIANN appears less reliable for West Africa, as it shows a trend toward reduced heavy rainfall during the first rainy season over recent decades—a pattern that diverges from the increases in heavy rainfall recorded by rain gauge observations in southern Ghana from 1970 to 2014 (59).

The upper tails of the CHIRPS and PERSIANN daily rainfall distributions are compressed relative to Ghanaian rain gauge observations (*SI Appendix*, Fig. S9). Comparing April–June daily rainfall distributions (days ≥ 1 mm, 2000–2024), CHIRPS, PERSIANN, and ERA5 reanalysis produce peaked distributions concentrated below ∼5 mm d^−1^ with thin upper tails, whereas TAMSAT exhibits a broader distribution, with greater density in the 10 to 20 mm d^−1^ range, and aligns most closely with the rain gauge data. Ref. 35 reaches a similar conclusion in an independent validation against 38 rain-gauge stations in Ghana and Zambia: TAMSAT achieves the highest probability of detection for moderate daily rainfall, between 50% and 80% across nearly all Ghanaian stations, while all three products have low skill for heavy and violent rain. Because our wet-season predictor is the 95th percentile of daily rainfall, products that compress the upper tail reduce year-to-year contrast and bias the coefficient toward zero. We therefore treat TAMSAT as the most appropriate product for this analysis.

#### Dry-season rainfall metric in Ghana and Ecuador.

For Ghana, we quantify dry-season rainfall as the daily-mean precipitation across the four climatologically driest months of the year, November to February of the following calendar year. We opt for the mean rather than a low percentile (e.g., the 5th percentile) because the prevalence of zero-rainfall days limits the informativeness of low percentiles.

For Ecuador, we quantify dry-season rainfall as the daily-mean precipitation across the four climatologically driest months of the year, June–September, prior to the harvest year. In addition to mean dry-season rainfall and mean soil moisture, we consider dry-day counts and maximum dry-spell duration. These metrics are in keeping with previous work (14). Dry-day counts are calculated as the monthly number of days with daily precipitation below 1 mm d^−1^, characterizing dry-spell frequency. Consecutive dry days is defined as the maximum number of successive days with daily precipitation below 1 mm d^−1^ within each month, representing drought persistence.

### Yield Data Processing.

#### Ghana subnational cocoa production data.

For Ghana, we use district-level annual cocoa production data provided by the Ghana Cocoa Board from the 2000/2001 to 2022/2023 seasons. We estimate the 2023/2024 season anomaly—whose shortfalls drove large global price increases beginning in 2024—based on national production data from the USDA Ghana Cocoa Report (9) as a 20% reduction relative to the prior season; this inferred value is shown as a dotted line in Fig. 2*B*.

Data for 68 districts were provided by the Ghana Cocoa Board. Two districts contained no data (Akim Akoasa and Bodi), resulting in 66 districts used in the analysis as shown in Fig. 1 *B* and *C*.

To isolate interannual deviations in productivity from long-term trends, such as in area harvested, we first fit a linear trend to each district’s total annual production and then divide the production values by that trend. Tarkwa and Sunyani are the only districts for which the linear detrending gives an outlier for the first value in 2000/2001 (e.g., below −300 for Tarkwa) and therefore we instead take the logarithm, subtract a linear trend, and then exponentiate to recover fractional anomalies. Because cocoa trees require three to five years to bear fruit and can be productive for about 40 y, the interannual variations in planted area are much smaller than for annual field crops. Dividing by the trend yields a “fractional production” time series, a proxy for yields in the absence of reliable area harvested data over time per district.

Regarding uncertainties in area harvested and thus yields, in Ghana, ref. 64 recently highlighted a 30% discrepancy between their estimate of area harvested, derived from Sentinel satellite data and a machine-learning approach, and the estimate reported by the Ghana Cocoa Board. Uncertainty in area harvested primarily affects the absolute magnitude and low-frequency variability of reported yields. Year-to-year noise in area estimates may also contribute to additional variability in fractional production; however, such noise is expected to be largely uncorrelated with climate variability and would therefore reduce statistical power rather than systematically bias inferred climate–yield relationships.

#### Ecuador and Indonesia: FAO national cocoa production, area harvested, and yield data.

For Ecuador and Indonesia, we do not have access to reliable subnational yield data and instead use national-level statistics from the Food and Agriculture Organization of the United Nations (FAO), accessed via the FAOSTAT database. The FAO provides cocoa production, area harvested, and yield data at annual resolution. We use FAO yield.

FAO cocoa statistics are reported by calendar year, whereas cocoa growing seasons can span two calendar years (*SI Appendix*, Table S1). The FAO methodology assigns production to the calendar year in which the harvest, or the bulk of it, occurs, but does not specify how this rule is applied for cocoa in each country. We therefore infer the alignment separately for each country. For Ecuador, the main harvest spans October–January, and the bulk falls in the first of these two calendar years. We take the FAO year to correspond to the year in which the harvest begins, matching February–April rainfall in calendar year 2000 to FAO yield listed as 2000. For Indonesia, cocoa is harvested nearly year-round, but Indonesian production is compiled on an October–September marketing year, and in Sulawesi the main harvest begins in late September, following the April–June rains by roughly 5–6 months. The crop set by the April–June rains of year t is therefore harvested across the October(t)–September(t+1) marketing year and reported under the following calendar year; we accordingly match April–June rainfall in year t to the FAO yield listed for year t+1 (e.g., April–June 2000 rainfall to yield listed as 2001).

For Ecuador, national-level cocoa yield data span 1961–2021. There is no strong linear trend from approximately 1961–2010, after which a clear breakpoint emerges and yields begin increasing linearly (*SI Appendix*, Fig. S3 *B* and *D*). A breakpoint is objectively identified in 2009 as the first year in which yields exceed the 1961–2010 average. We therefore fit linear trends separately over the periods 1961–2009 and 2010–2021 and divide each segment by its respective trend. Results are robust to the precise choice of inflection year.

The observed yield inflection likely reflects multiple nonclimatic factors, including expansion into more productive areas, improved agricultural practices, and in particular the adoption of higher-yielding and more disease-resistant varieties, notably CCN-51 (65, 66). Following the severe 1997–98 El Niño, which strongly diminished yields (Fig. 2*D*), the more resilient CCN-51 cacao was increasingly prioritized over heirloom varieties, and by 2017 accounted for approximately 70% of national production (66). While heirloom cacao varieties are celebrated for their flavor profiles, they typically have lower yields and greater susceptibility to fungal diseases such as witches’ broom and frosty pod rot.

As an alternative approach, we also consider removing low-frequency variability using a high-pass filter, which suppresses multidecadal changes associated with nonclimatic factors such as cultivar adoption and management improvements while retaining year-to-year fluctuations. This filtering yields interannual yield anomalies that are highly similar to those obtained from piecewise linear detrending and leads to the same inferred climate–yield relationships. These consistent results indicate that our findings are not sensitive to the specific method used to remove low-frequency variability from the yield time series.

For Indonesia, the UN FAO data span 1960–2020. We focus on the period 1983–2010, during which the satellite rainfall record begins (1983–2024), and the relationship between production and harvested area appears approximately linear. After 2010, this relationship breaks down, with data points forming a nonlinear cluster (*SI Appendix*, Fig. S3*C*). There are also more clusters of nearly identical and repeated yield values evident in the time series after 2008, which may reflect reporting artifacts. The decline in cocoa yields in Indonesia in the 2000s (*SI Appendix*, Fig. S3*A*) has been linked to farmers replacing cocoa trees with more profitable crops such as oil palm or cloves (38). This shift has been attributed to a combination of economic and agronomic pressures: persistently low global cocoa prices and rising production costs due to severe infestations of cocoa pod borer, a type of moth, and the added burden of fungal diseases such as black pod and vascular streak dieback. While new cocoa-growing regions have emerged, their development has not been sufficient to offset the decline in Sulawesi (38). To account for these long-term shifts, we fit a linear trend to yield data from 1983–2010 and divide by that linear trend to obtain fractional yield data. Ending in 2008 or 2009 improves model fit, but we end in 2010 for simplicity.

### Phenological Alignment.

#### Phenological cycle and harvest year definition.

For Ghana, the April–June window coincides with the main flowering and early pod development period, when rainfall exerts the strongest influence on floral and cherelle production (29). Pods initiated during this period contribute primarily to the main cocoa harvest, which normally extends from October through March, with recent seasons frequently extending the main crop through May; 70 to 80% of annual production is harvested during this main crop season (9). This implies a lag of approximately 4 to 8 mo between wet-season climate exposure and harvest, supporting typical cocoa pod maturation times of approximately 5 to 6 mo from pollination to harvest stage.

For Ecuador, production regions experience a primary rainy season from approximately December to May, peaking in February–April, during which there is flowering and early pod development. We therefore define the wet-season extreme-rainfall metric using February–April and assign these exposures to the production year of the subsequent harvest. Although cocoa is harvested throughout much of the year in Ecuador, the largest share of national production occurs during the late harvest period, approximately October–January, corresponding to a lag of roughly 6 to 8 mo from flowering to harvest.

For Indonesia (Sulawesi), the main rainy season occurs during April–June, overlapping peak flowering and early pod development, while the principal harvest extends from approximately September through December. This again implies a lag of about 5 to 6 mo between wet-season rainfall extremes and harvests.

Across all three regions, the lag between wet-season climate exposure and the main harvest is consistent with the typical cocoa pod development time of approximately six months from successful pollination to pod maturity. Accordingly, climate windows are interpreted as acting on vulnerable phenological stages that determine the number of pods available for harvest, with windows mapped to the production year in which the associated pods are harvested.

### Statistical Methods.

#### Regularized regression and random forest models.

All candidate predictors (*SI Appendix*, Table S3) were standardized and included simultaneously in penalized linear regressions and the random forest. Regularization hyperparameters were tuned using time-series-aware cross-validation with expanding training windows (*TimeSeriesSplit*), such that model fitting and variable selection were based exclusively on past data. Lasso and elastic net regressions were implemented with cross-validated regularization strength (*LassoCV* and *ElasticNetCV*; for elastic net, the L1/L2 mixing parameter was also cross-validated). Standardization makes coefficient magnitudes comparable across predictors. Random forest models were trained using the same cross-validation splits, and predictor importance was evaluated using impurity-based importance metrics averaged across folds.

Regarding the features, we note that more data-driven representations of weather variability, such as principal component analysis of the rainfall and temperature time series, could capture additional structure. While our results indicate that simple seasonal aggregates already explain a substantial fraction of interannual yield variability and offer clearer interpretability for adaptation-relevant insights, exploring higher-dimensional embeddings derived from deep learning approaches may be a useful direction for future work.

#### Nonlinear temperature and rainfall model.

It is possible to examine more general and flexible models of cocoa yield sensitivity to environmental conditions. We explore versions of a nonlinear regression of daily weather over the growing season that give results that are consistent with our linear model.

Considering Ghana, the wet season is selected as April–June, and for every grid cell and every day, we include the mean daily rainfall, W, its square $W^{2}$ and its cube $W^{3}$ and then sum across each grid cell and day; this yields the three time series $\left(W_{t},W_{t}^{2},W_{t}^{3}\right)$. Squared and cubic terms allow for nonlinear responses. The dry-season aggregate spans November(t)–February(t+1). Because the cocoa growing season straddles two calendar years, November–December are assigned to the current year, whereas January–February are counted in the preceding year so that each dry season is labeled by the year in which it begins (e.g., the November 2000-February 2001 mean is labeled as 2000). The same procedure gives three time series $\left(D_{t},D_{t}^{2},D_{t}^{3}\right)$ for each season-year t. As for the linear model, we consider median detrended cocoa production (fractional production) across districts, $Y_{t}$, which is joined with the wet- and dry-season aggregates, giving 23 observations (2000/2001–2022/2023 seasons) with one response variable, six rainfall predictors, a year (t), and an intercept, $\beta_{0}$. In this case we end in the 2022/2023 season because global daily land maximum, mean, and minimum temperature data from Berkeley Earth (31) were only available through 2023. An ordinary-least-squares regression is then fitted,

[2] $$\begin{array}{c} Y_{t}=\beta_{0}+\beta_{1}W_{t}+\beta_{2}W_{t}^{2}+\beta_{3}W_{t}^{3}+\beta_{4}D_{t}+\beta_{5}D_{t}^{2}+\beta_{6}D_{t}^{3}+\beta_{7}t+\varepsilon_{t}. \end{array}$$

This method gives an $R^{2}=0.65$ and an adjusted $R^{2}=0.49$ and indicates a negative response to linear and cubic wet-season rainfall terms and a positive response to linear and quadratic dry-season rainfall terms. However, none of the individual coefficients are statistically significant at conventional levels (all P>0.05), supporting overfitting in a model that includes multiple higher-order terms.

Eq. **2** models fractional production or detrended yields, $Y_{t}$, as a nonlinear function of seasonal rainfall, with separate third-order polynomial terms for wet-season rainfall ($W_{t}$; April–June) and dry-season rainfall ($D_{t}$; November–February). The regression coefficients $\beta_{1}$–$\beta_{3}$ quantify the linear, quadratic, and cubic sensitivities of yield anomalies to wet-season rainfall, while $\beta_{4}$–$\beta_{6}$ represent the corresponding sensitivities to dry-season rainfall. The coefficient $\beta_{7}$ captures any remaining linear temporal trend not removed by detrending.

To aid interpretation of the nonlinear terms, *SI Appendix*, Fig. S6 shows the marginal rainfall effect obtained by varying either W or D over its empirical range while holding all other predictors fixed at their mean values. These marginal responses are given by

[3] $$\begin{array}{c} {\hat{Y}}_{\text{wet}}\left(W\right)={\hat{\beta }}_{1}W+{\hat{\beta }}_{2}W^{2}+{\hat{\beta }}_{3}W^{3},\;{\hat{Y}}_{\text{dry}}\left(D\right)={\hat{\beta }}_{4}D+{\hat{\beta }}_{5}D^{2}+{\hat{\beta }}_{6}D^{3}. \end{array}$$

The marginal sensitivity to wet-season rainfall highlights a negative yield response to heavy April–June rainfall (*SI Appendix*, Fig. S6*A*).

Adding temperature predictors, also modeled as third-order polynomials for the same two seasons, leads to an $R^{2}=0.76$ but lowers the adjusted $R^{2}$ to 0.41, and raises AIC and BIC, indicating that including temperature does not improve the model. Various other versions of our nonlinear regression model including different polynomial degrees or interaction terms lead to qualitatively consistent albeit less skillful results.

If we pool data from Ghana, Sulawesi, and Ecuador for all available years and include a fixed effect for country, the model also continues to show a negative yield response to excessive rainfall during the wet season. The negative yield response is stronger for second-order than third-order polynomials, which may be an overfit model. The nonlinear regression models confirm that yield limitations are primarily associated with extreme rainfall during the wet season, but we focus on the linear representation as being a simpler representation of this dependence.

## Supplementary Material

Appendix 01 (PDF)

## Data Availability

Code for the statistical analyses and figure generation is available at https://zenodo.org/records/22032339 (67).
