# Supplementary material for: Rainfall extremes drive cocoa yield losses across the tropics
Source: Proc Natl Acad Sci U S A. 2026 Sep 14;123(39):e2606123123. doi: 10.1073/pnas.2606123123 (PMC13624598; doi:10.1073/pnas.2606123123)
Supplement: Supplementary file 1 [file pnas.2606123123.sapp.pdf]

PNAS

Supporting Information for

Rainfall extremes drive cocoa yield losses across the tropics

Anna Lea Albright, Rebecca Berkoh-Oforiwaa and Peter Huybers

Anna Lea Albright.
E-mail: annaleaalbright@fas.harvard.edu

This PDF file includes:

Figs. S1 to S9
Tables S1 to S7
SI References

Tables

Table S1. Three rainiest months, dry-season rainfall, and approximate cocoa harvest times for selected regions. According to the International Cocoa Organization (ICCO) (1), the most pronounced gap between the main and secondary (or mid-crop) harvests occurs in Africa, where the mid-crop represents roughly 15%–20% of total production; elsewhere, the distinction between the two harvest periods is less marked.

Region	Rainiest Months	Dry Season Rainfall	Main Harvest	Secondary Harvest
Ghana	April–June	November–February	October–March/May	May–August
Sulawesi	April–June	—	September–December	March–July
Ecuador	February–April	June–September	October–December	March–June

Table S2. Daily-mean precipitation datasets used in this study.

Name	Full name	Description	Time period	Region
REGEN	Rainfall Estimates on a Gridded Network	Network of in-situ rain-gauge records (e.g. GHCN-Daily, GPCC); quality-controlled and interpolated with ordinary block kriging	1950–2016	Global land
PERSIANN	Precipitation Estimation from Remotely Sensed Information using Artificial Neural Networks	Infrared cloud-top brightness temperature (GridSat-B1); learns a temperature-rain relationship with ANN trained on NCEP Stage IV data, bias-adjusted with GPCP v2.2	1983–present	Global land/ocean
TAMSAT	Tropical Applications of Meteorology using SATellite data and ground-based observations	Infrared cloud-top brightness temperature (Meteosat) over Africa; uses a cold cloud duration threshold – the longer a pixel stays cold, the more rain is assumed; cloud top temperature threshold varies regionally and seasonally; calibrated with African rain-gauge archives	1983–present	Pan-African
CHIRPS	Climate Hazards Group InfraRed Precipitation with Station data	Blended precipitation product combining infrared cold cloud duration from geostationary satellites with in-situ rain-gauge observations; uses a climatology-based bias correction and smart interpolation to improve rainfall estimates in data-sparse regions	1981–present	Global land (50°S–50°N)

Table S3. Candidate climate predictors evaluated in various model formulations. All variables were computed at monthly resolution and tested in pairs of months unless otherwise noted. Soil moisture is included only for Ghana given that it is derived from an African-only data set, TAMSAT.

Predictor	Description
Mean rainfall	Monthly mean of daily precipitation (mm/day), representing average water availability.
Extreme rainfall	Monthly 95th percentile of daily precipitation (mm/day), characterizing the upper tail of rainfall intensity and heavy rainfall events.
Mean soil moisture	Monthly mean root-zone soil moisture availability (β; 0–100), a unitless plant moisture stress factor derived from the TAMSAT-JULES land surface model (2).
Dry day count	Monthly number of days with daily precipitation < 1 mm/day, representing dry-spell frequency.
Consecutive dry days	Maximum number of consecutive dry days with daily precipitation < 1 mm/day, per month, representing drought persistence.
Cold temperature exposure	Cumulative sum of daily minimum temperature below 18°C within each month (degree-days), representing cold stress, following physiological thresholds given in (1).
Heat exposure	Cumulative sum of daily maximum temperature above 32°C within each month (degree-days), representing heat stress, following physiological thresholds given in (1).

Feature	LASSO	ENet	RF imp.	|ENet|	LASSO sel	ENet sel	RF sel
rain_q95_0506	-0.0298	-0.0219	0.0464	0.0219	Y	Y	Y
sm_c4grass_mean_0102	0.0389	0.0181	0.0883	0.0181	Y	Y	Y
tmax_edd32_0910	0.0170	0.0166	0.0559	0.0166	Y	Y	Y
tmax_edd32_0304	0.0084	0.0142	0.0326	0.0142	Y	Y	N
sm_c4grass_mean_1112	0.0107	0.0139	0.0814	0.0139	Y	Y	Y
rain_q95_0102	0.0103	0.0120	0.0868	0.0120	Y	Y	Y
rain_mean_0102	0.0000	0.0118	0.0668	0.0118	N	Y	Y
rain_mean_1112	0.0000	0.0089	0.0887	0.0089	N	Y	Y
rain_mean_0506	-0.0000	-0.0024	0.0718	0.0024	N	Y	Y
rain_mean_0910	-0.0000	-0.0000	0.0857	0.0000	N	N	Y

Table S4. For Ghana, using TAMSAT daily rainfall and Berkeley Earth daily temperatures over the 23 observed seasons (2000/01–2022/23, $n = 23$; the daily temperature record ends in 2023), the top-ranked climate features and whether they were selected by each statistical learning model: Least Absolute Shrinkage and Selection Operator (LASSO), elastic net regression (ENet), and Random Forest (RF). Lasso and elastic net values correspond to standardized regression coefficients, while RF importance denotes mean decrease in impurity averaged across cross-validation folds. Features are sorted by the absolute value of the elastic net coefficient. Selection flags indicate nonzero coefficients for Lasso and elastic net, and top-K importance for RF, where K equals the number of elastic net–selected features. Feature names follow the predictor definitions in Table S3 and encode both variable type and month pair. For example, `rain_q95_0506` denotes extreme rainfall defined as the 95th percentile of daily precipitation averaged over May–June; `rain_mean_0102` denotes mean rainfall over January–February; `sm_c4grass_mean_0102` denotes mean root-zone soil moisture over January–February; and `tmax_edd32_0910` denotes cumulative degree-days of daily maximum temperature above 32°C over September–October.

Feature	Lasso	ENet	RF imp.	|ENet|	Lasso sel	ENet sel	RF sel
rain_q95_0304_cur	-0.0642	-0.0573	0.1534	0.0573	Y	Y	Y
rain_q95_0102_cur	-0.0508	-0.0316	0.0692	0.0316	Y	Y	Y
rain_drycount_0708_prev	-0.0269	-0.0313	0.0183	0.0313	Y	Y	N
cold_excess_0304_cur	0.0193	0.0261	0.0772	0.0261	Y	Y	Y
rain_drycount_0102_cur	0.0000	0.0239	0.0192	0.0239	N	Y	N
rain_mean_0102_cur	-0.0000	-0.0207	0.0454	0.0207	N	Y	Y
rain_drycount_0304_cur	0.0070	0.0190	0.0204	0.0190	Y	Y	N
cold_excess_1112_prev	0.0000	0.0187	0.0783	0.0187	N	Y	Y
rain_mean_0708_prev	0.0009	0.0183	0.0293	0.0183	Y	Y	Y
rain_mean_0304_cur	-0.0179	-0.0156	0.0570	0.0156	Y	Y	Y
rain_drycount_0910_prev	-0.0000	-0.0151	0.0465	0.0151	N	Y	Y
hot_excess_0102_cur	-0.0000	-0.0141	0.0256	0.0141	N	Y	Y

Table S5. For Ecuador, using REGEN daily rainfall (1983–2016) and Berkeley Earth daily temperatures, the top-ranked climate features and whether they were selected by each statistical learning model: Least Absolute Shrinkage and Selection Operator (Lasso), elastic net regression (ENet), and Random Forest (RF). Lasso and elastic net values correspond to standardized regression coefficients, while RF importance denotes mean decrease in impurity. Features are sorted by the absolute value of the elastic net coefficient. Selection flags indicate nonzero coefficients for Lasso and elastic net, and top-K importance for RF, where K equals the number of elastic net–selected features. Feature names follow the predictor definitions in Table S3 and encode both variable type and month pair (e.g., `0304` denotes March–April, and `cur`/`prev` indicate the current year (e.g., harvest year) or previous year).

**Model**	n	k	**Adj.** R^2	**AIC**	**BIC**	$\triangle$Adj. R^2	$\triangle$AIC	$\triangle$BIC
Baseline: rain q95 Apr–Jun + rain mean Nov–Feb	24	2	0.65	-34.30	-30.76	0.00	0.00	0.00
+ `tmax_edd32_0910`	24	3	0.64	-32.86	-28.14	-0.01	1.44	2.62
+ `tmax_edd32_0910` + `tmax_edd32_0304`	24	4	0.65	-32.93	-27.04	0.00	1.37	3.73

Table S6. Model comparison for Ghana using standardized predictors and identical sample years ($n = 24$, number of predictors k). The baseline model includes wet-season rainfall extremes (April–June 95th percentile) and dry-season mean rainfall (November–February). Additional models incrementally include temperature-based stress metrics (cumulative degree-days above 32°C; see Table S4). Differences ($\triangle$) are computed relative to the baseline specification.

Region	ENSO (Niño index)		Atlantic Niño		Trend in ENSO–residuals	
	Box & months	R^2	Box & months	R^2	β (mm day^{-1} yr^{-1})	P
Ghana	3.4 (March–May)	0.41	ATL3 (Feb–May)	0.21	0.05	0.03
Côte d'Ivoire	3.4 (March–May)	0.46	ATL3 (Feb–May)	0.30	0.05	0.03
Cameroon	3.4 (Aug–Oct)	0.01	ATL3 (Sep–Nov)	0.34	0.06	0.04
Ecuador	1+2 (Feb–Apr)	0.42	—	—	-0.11	0.02
Sulawesi	3.4 (Apr–Jun)	0.07	—	—	0.05	0.09
Bahia	1+2 (Oct–Dec)	0.13	—	—	-0.04	0.32

Table S7. ENSO and Atlantic Niño influences on wet-season rainfall extremes. The ENSO columns give the variance (R^2) in the peak wet-season 95th-percentile daily rainfall explained by regression on the standardized Niño sea surface temperature index (1+2 or 3.4) over 1983–2023, for the region and Niño-index months indicated. For Ghana, Côte d'Ivoire, and Cameroon the Niño index precedes the rainfall season by one month; for the other regions the same months are used. The Atlantic Niño (ATL3) columns give the corresponding R^2 for the West African regions. Both the Niño and Atlantic Niño indices are linearly detrended to remove the long-term warming trend before regression. The trend columns report the slope β (mm day^{-1} yr^{-1}) and P-value of the linear trend in the ENSO-residual rainfall over 1983–2023. For Sulawesi, the April–June cocoa season is used, as for the other regions; ENSO explains little of the April–June rainfall variance there ($R^2 \approx 0.07$), although it strongly modulates October–December rainfall ($R^2 \approx 0.62$), in a different season. Index regions: Niño 1+2 = $10°$S–$0°$, $90°$W–$80°$W; Niño 3.4 = $5°$N–$5°$S, $170°$W–$120°$W; ATL3 = $3°$S–$3°$N, $20°$W–$0°$.

Figures

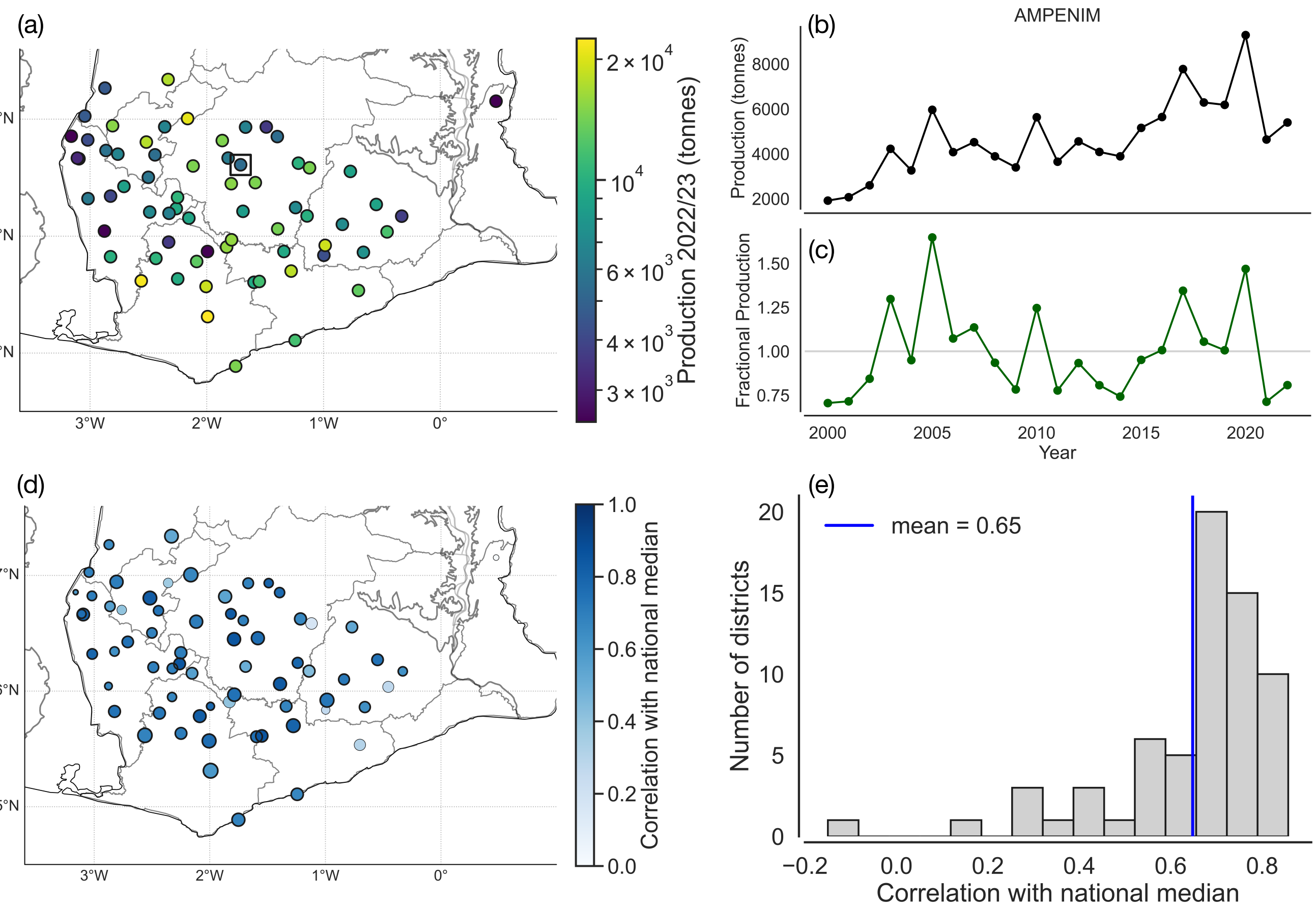

Fig. S1. (a) Absolute cocoa production by district in 2022 (tonnes). The district outlined by the black box is presented in the time series in panel (b) of annual cocoa production from the 2000/2001 to 2022/2023 growing seasons. (c) Fractional (detrended) production for the same district, obtained by dividing the raw production time series by its linear trend. (d) Spatial distribution of the Pearson correlation coefficient between district-level fractional production time series (2000/2001 to 2022/2023) and the national median fractional production time series shown in Fig. 2b of the main manuscript. The marker size is proportional to production in the 2022/2023 growing season. (e) Histogram of these correlations of individual districts with the national median, with the blue vertical line indicating the mean value.

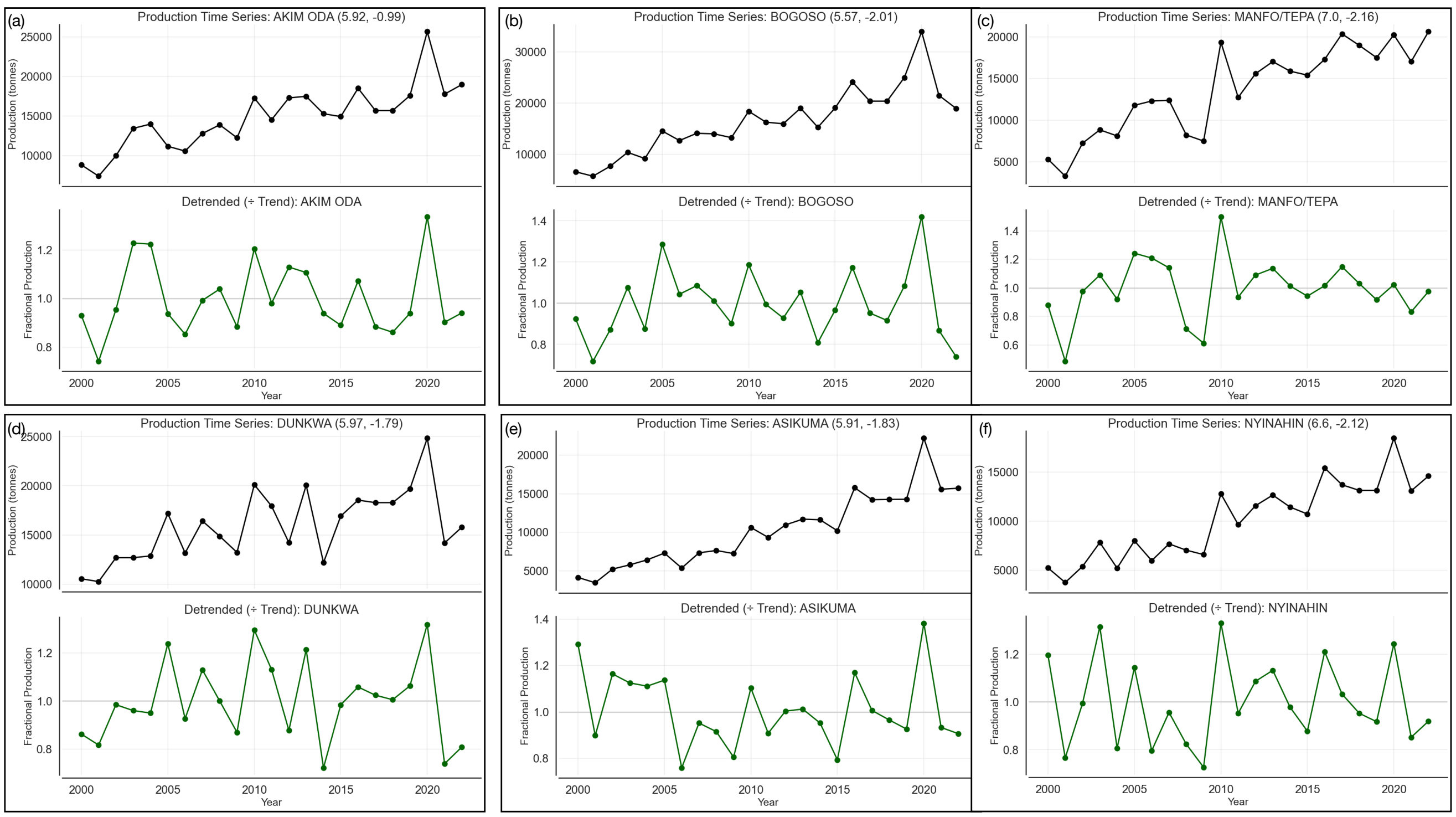

Fig. S2. Further examples of absolute production (in tonnes) per district and detrended 'fractional production' for six additional high-producing districts.

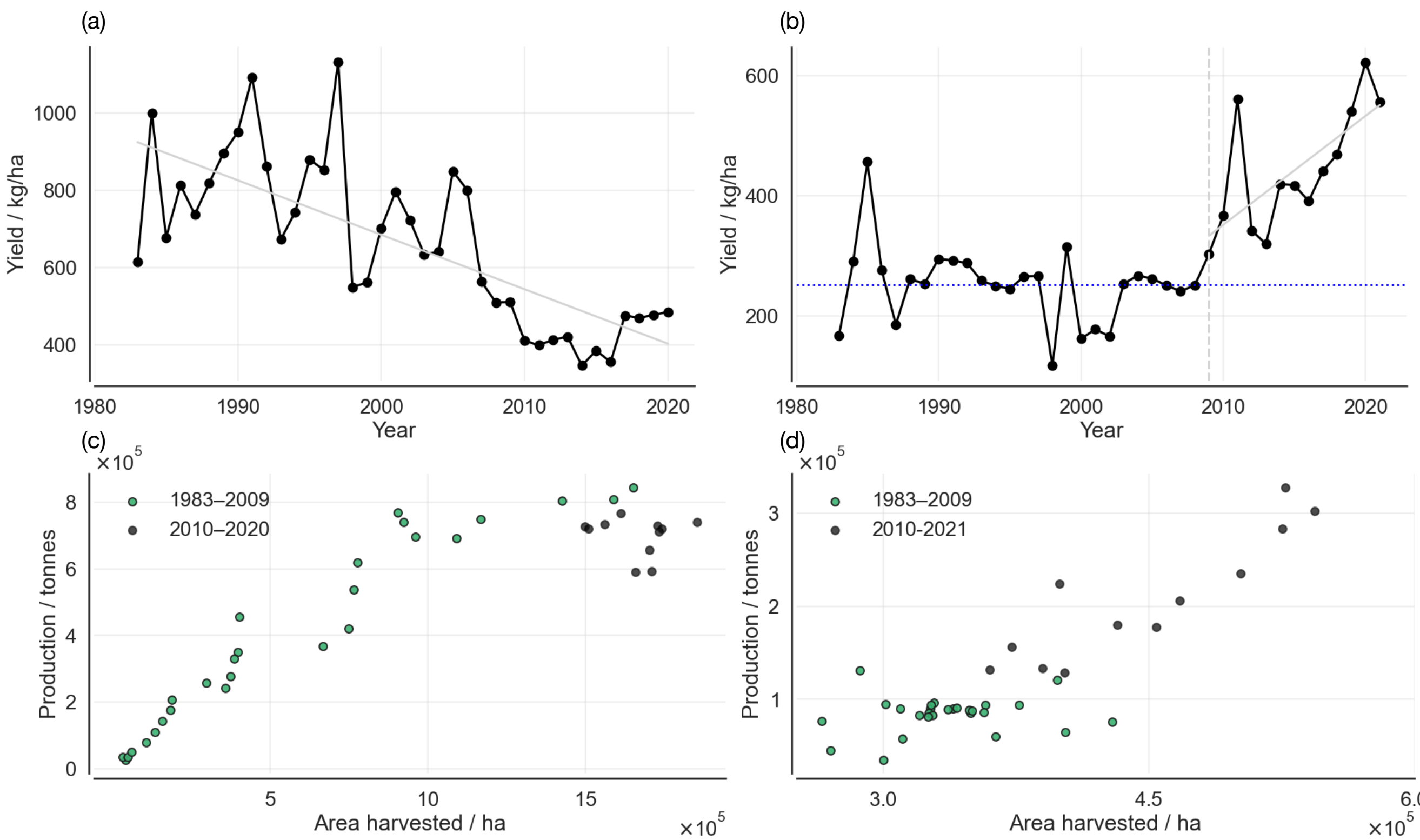

Fig. S3. Time series of UN FAO yield data for Indonesia (a) and Ecuador (b) from 1983 to the last year available, and scatter plots of production (tonnes) versus area harvested (ha) again for Indonesia (c) and Ecuador (d).

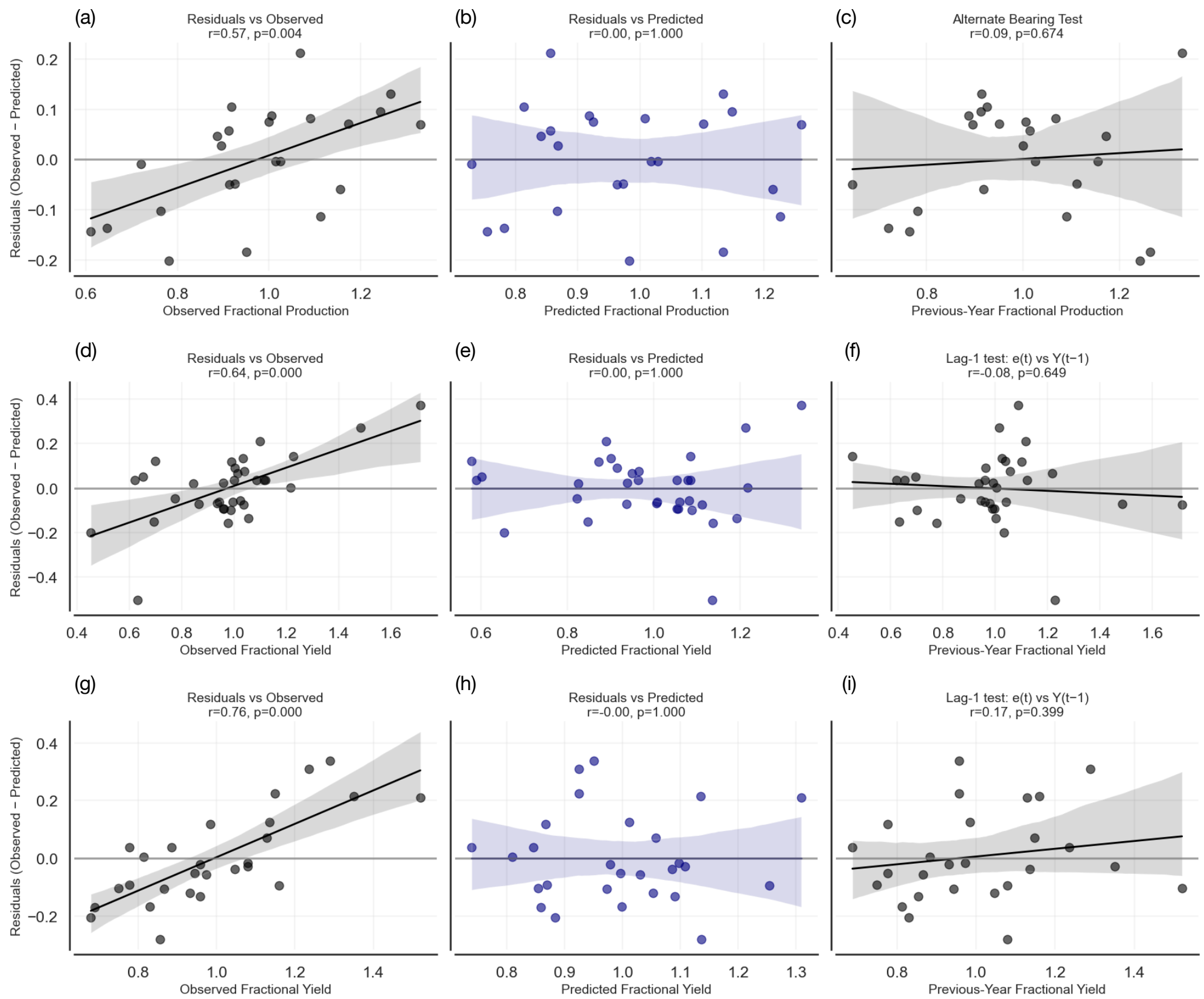

Fig. S4. Residual diagnostics for the baseline linear rainfall model applied to Ghana (a–c), Ecuador (d–f), and Sulawesi, Indonesia (g–i). Residuals are defined as observed minus predicted fractional production (Ghana) or detrended yield (Ecuador and Sulawesi). Panels (a,d,g) show residuals versus observed values, testing for state-dependent bias; panels (b,e,h) show residuals versus predicted values, testing for heteroscedasticity or functional misspecification; panels (c,f,i) show residuals versus previous-year observed production (lag-1 test), evaluating potential alternate bearing or carry-over effects. Reported statistics indicate Pearson correlation coefficients and P-values. Across regions, residuals exhibit no significant association with predicted values and no consistent lag-1 dependence, indicating that model errors do not reflect systematic alternate bearing effects or temporal persistence.

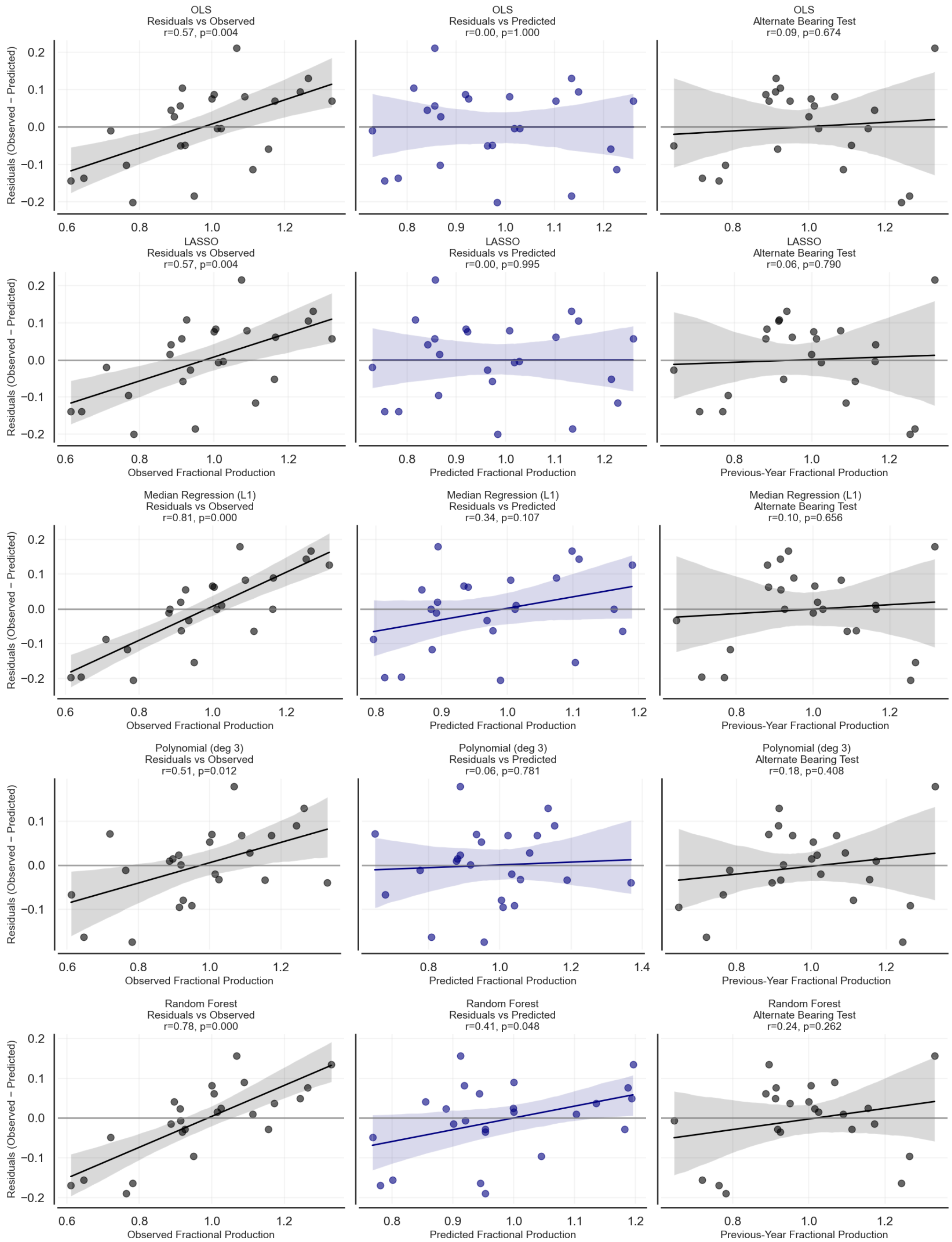

Fig. S5. Residual diagnostics for alternative model formulations applied to Ghana, corresponding to the analyses described in the main text. Rows show results for ordinary least squares (OLS), lasso regression, a median (L1) loss, third-order polynomial regression, and random forest models. Columns show residuals versus observed fractional production (left), residuals versus predicted values (center), and residuals versus previous-year fractional production (right; alternate bearing test). Residuals are defined as observed minus predicted fractional production. Pearson correlation coefficients and P-values are indicated in each panel. Across linear, regularized, nonlinear, and tree-based formulations, residual structure is broadly similar, with no significant lag-1 dependence. These results indicate that underestimation of the most positive and negative anomalies is not an artifact of functional form or loss function and does not reflect systematic alternate bearing dynamics.

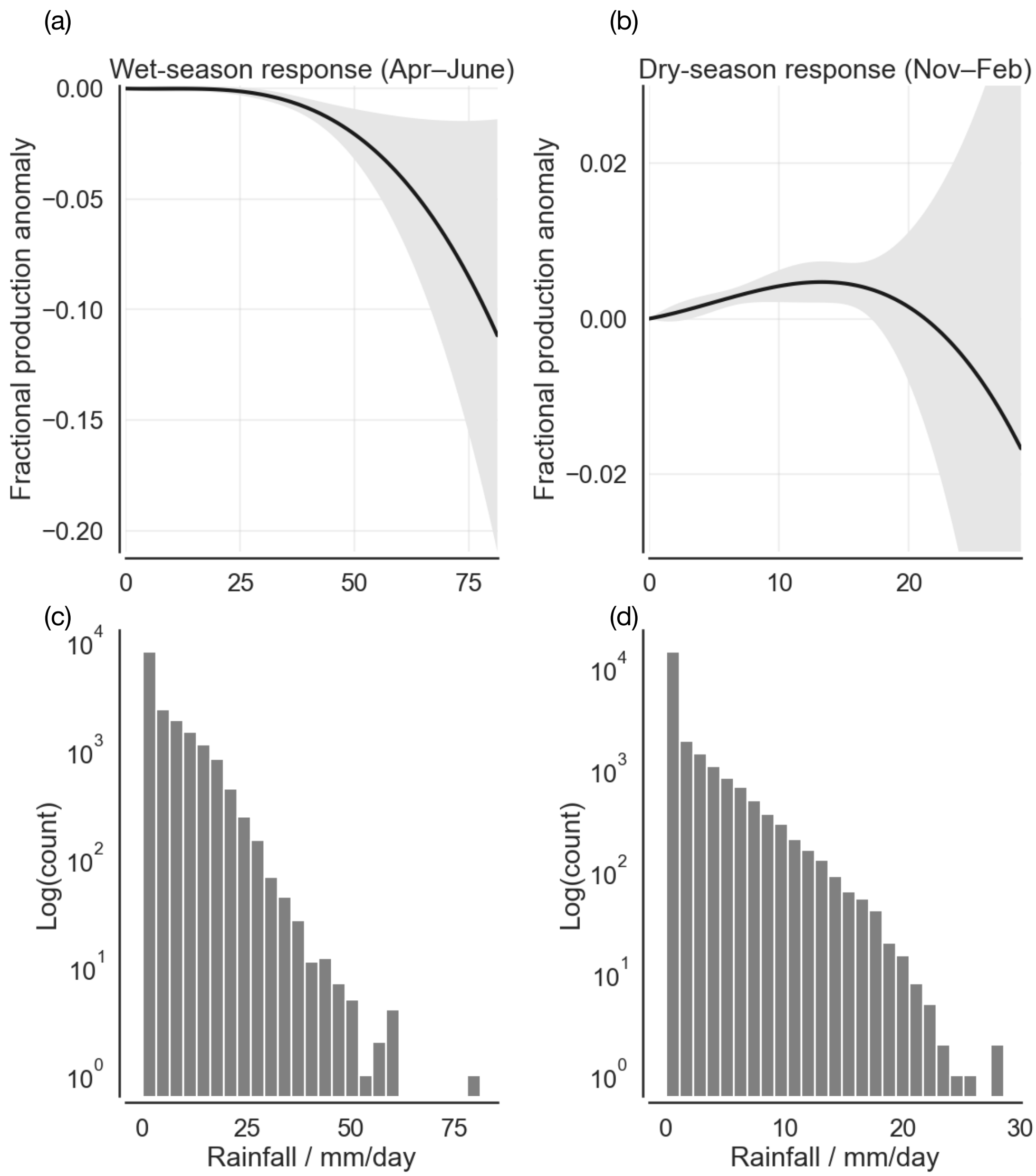

Fig. S6. Nonlinear model results for Ghana for daily rainfall, with marginal sensitivity shown for wet-season rainfall (a) and dry-season rainfall (b), with corresponding logarithmic histogram counts (c,d), highlighting which parts of the curve are well informed by data (dense histogram mass) and which rely on extrapolation (sparse tails). An 80% confidence band is drawn as $\hat{Y} \pm 1.28\ \mathrm{SE}$, the standard error.

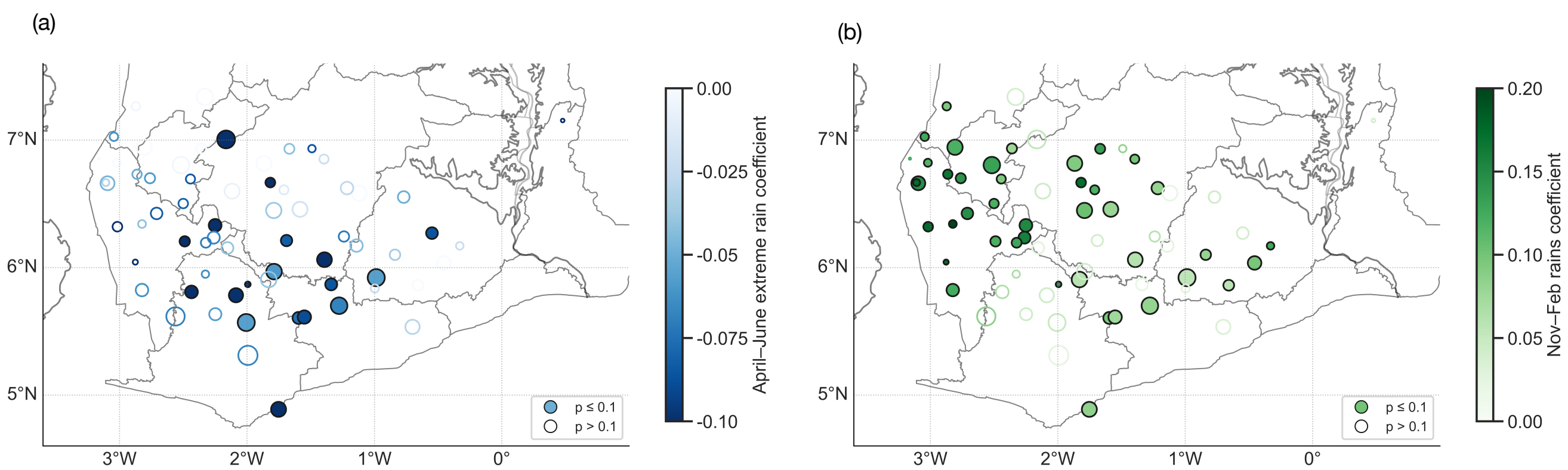

Fig. S7. Analysis of district-level cocoa production anomalies, as in Fig. 3 in the main manuscript, but using TAMSAT rainfall data at 0.25x0.25° instead of 1x1° resolution. Linear regressions are repeated using nearest-neighbor rainfall values for each district with 0.25x0.25° TAMSAT rainfall data; panels show the estimated coefficients for heavy rainfall (left) and for mean dry-season rainfall (right) across 66 Ghanaian districts. Filled markers indicate statistical significance at the 10% level ($P \leq 0.1$); open markers indicate $P > 0.1$. The marker size is proportional to 2022/2023 production in tonnes. Using these higher-resolution data, 71% of the northern records of fractional production anomalies show moderately significant associations ($P < 0.1$) with dry-season rainfall, and below 6°N, 45% of districts show such significance. Conversely, 55% of districts in the south are significant with respect to extreme rainfall, but only 16% in the north.

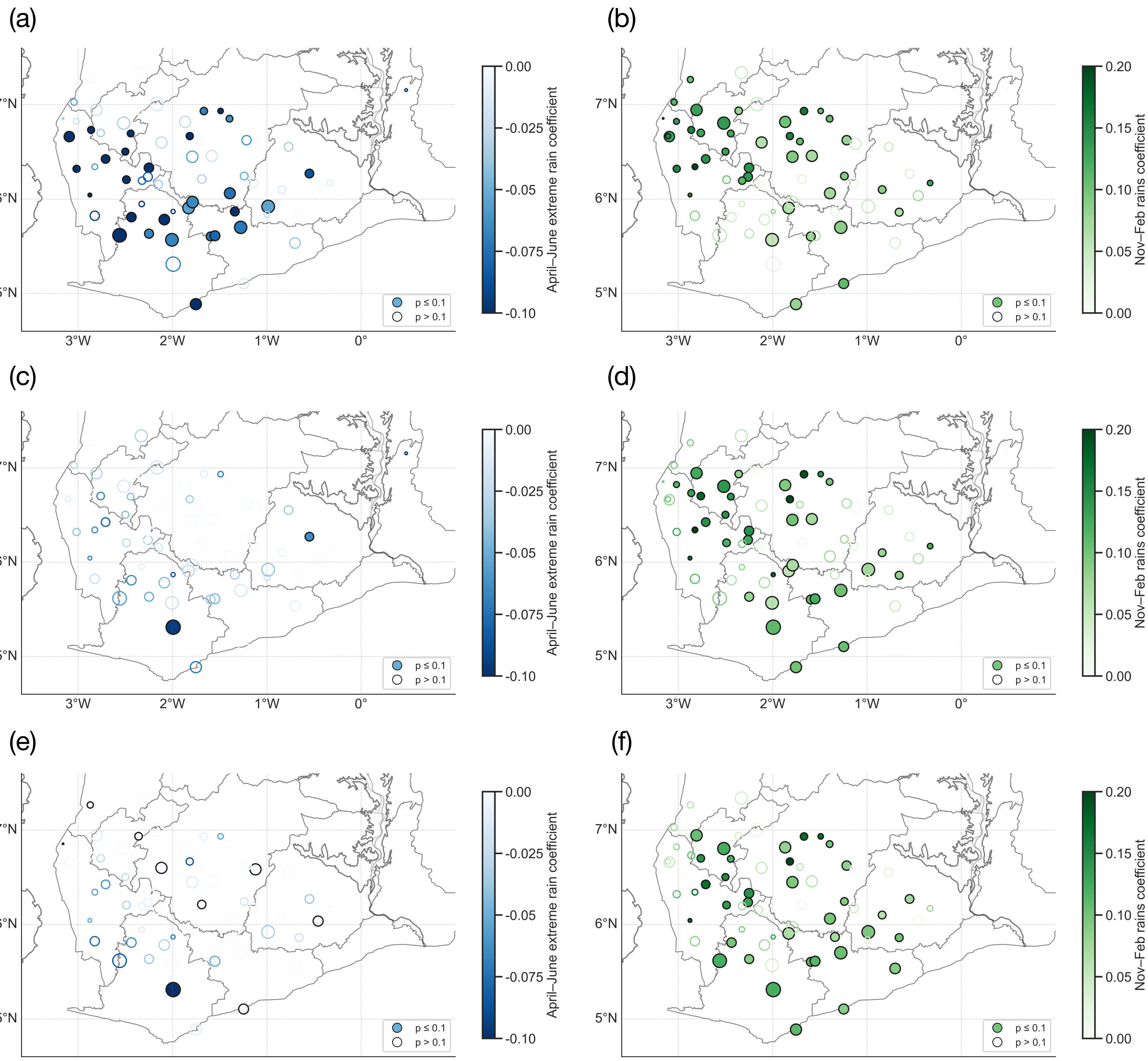

Fig. S8. Comparison of district-level regression coefficients across three rainfall products: TAMSAT (a, b), CHIRPS (c, d), and PERSIANN (e, f), all at 1x1° resolution. Linear regressions are repeated for each district using nearest-neighbor rainfall values from each product, with panels showing estimated coefficients for April–June extreme rainfall (left column; a, c, e) and November–February mean dry-season rainfall (right column; b, d, f) across 66 Ghanaian districts. Filled markers indicate statistical significance at the 10% level ($P \leq 0.1$); open markers indicate $P > 0.1$. Marker size is proportional to 2022/2023 production in tonnes.

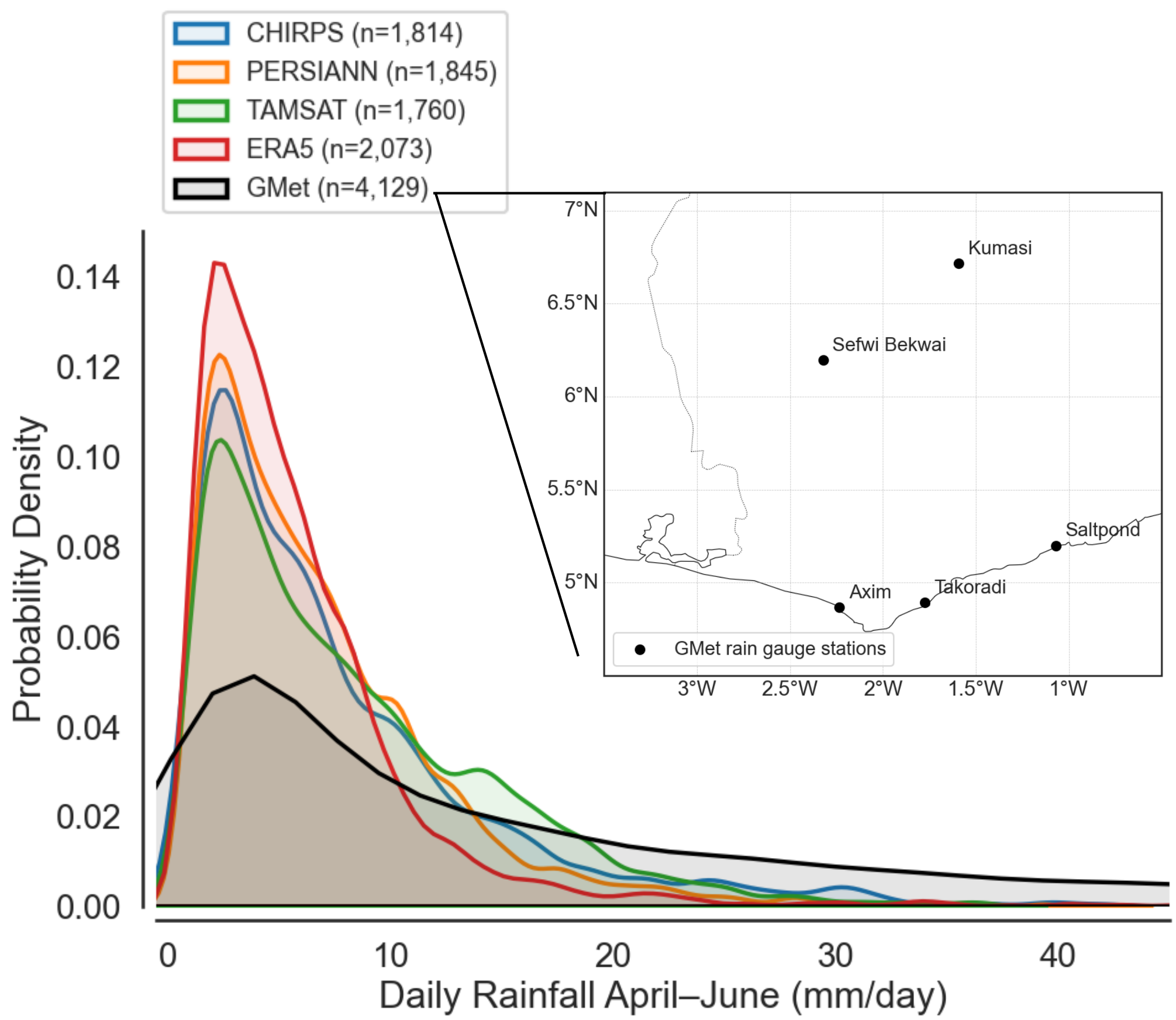

Fig. S9. Daily rainfall for April to June from 2000 to 2024, except for PERSIANN for which we have data through 2023 (e.g., April-June 2023 rainfall for the Ghana 2023/2024 harvest season, our last Ghana cocoa year). The 'n' in the legend refers to the number of rainy days for these months over the time period. GMet refers to rain gauge station data from the Ghana Meteorological Agency, pooled (not time-averaged per day across stations), hence the larger number of rainy days compared to the gridded products. Time-averaging across stations gives comparable results. The inset shows the location of the five rain gauges.

References

1. International Cocoa Organization, Growing Cocoa (https://www.icco.org/growing-cocoa/) (2023).
2. RI Maidment, et al., A new, long-term root zone soil moisture dataset for operational agricultural drought monitoring over Africa. *Sci. Data* (2026).
